# Age‐dependent loss of hepatic SIRT1 enhances NLRP3 inflammasome signaling and impairs capacity for liver fibrosis resolution

**DOI:** 10.1111/acel.13811

**Published:** 2023-03-31

**Authors:** Jennifer Adjei‐Mosi, Qing Sun, Steven Blake Smithson, Gavyn Lee Shealy, Krupa Dhruvitha Amerineni, Zerong Liang, Hanqing Chen, Mei Wang, Qinggong Ping, Jingyan Han, Masahiro Morita, Amrita Kamat, Nicolas Musi, Mengwei Zang

**Affiliations:** ^1^ Barshop Institute for Longevity and Aging Studies, Center for Healthy Aging San Antonio Texas USA; ^2^ Department of Molecular Medicine The University of Texas Health San Antonio San Antonio Texas USA; ^3^ Boston University School of Medicine Boston Massachusetts USA; ^4^ Geriatric Research, Education and Clinical Center South Texas Veterans Health Care System San Antonio Texas USA

**Keywords:** aging, alcohol‐associated liver disease, hepatocyte‐specific SIRT1 knockout, hepatic stellate cells (HSCs), liver fibrosis, MCC950, NLRP3 inflammasome

## Abstract

Our studies indicate that the longevity factor SIRT1 is implicated in metabolic disease; however, whether and how hepatocyte‐specific SIRT1 signaling is involved in liver fibrosis remains undefined. We characterized a functional link of age‐mediated defects in SIRT1 to the NLRP3 inflammasome during age‐related liver fibrosis. In multiple experimental murine models of liver fibrosis, we compared the development of liver fibrosis in young and old mice, as well as in liver‐specific SIRT1 knockout (SIRT1 LKO) mice and wild‐type (WT) mice. Liver injury, fibrosis, and inflammation were assessed histologically and quantified by real‐time PCR analysis. In a model of hepatotoxin‐induced liver fibrosis, old mice displayed more severe and persistent liver fibrosis than young mice during liver injury and after injury cessation, as characterized by inhibition of SIRT1, induction of NLRP3, infiltration of macrophages and neutrophils, activation of hepatic stellate cells (HSCs), and excessive deposition and remodeling of the extracellular matrix. Mechanistically, deletion of SIRT1 in hepatocytes resulted in NLRP3 and IL‐1β induction, pro‐inflammatory response, and severe liver fibrosis in young mice, mimicking the ability of aging to impair the resolution of established fibrosis. In an aging mouse model, chronic‐plus‐binge alcohol feeding‐induced liver fibrosis was attenuated by treatment with MCC950, a selective NLRP3 inhibitor. NLRP3 inhibition ameliorated alcoholic liver fibrosis in old mice by repressing inflammation and reducing hepatocyte‐derived danger signaling—ASK1 and HMGB1. In conclusion, age‐dependent SIRT1 defects lead to NLRP3 activation and inflammation, which in turn impairs the capacity to resolve fibrosis during aging.

## Graphical abstract

Deletion of SIRT1 in hepatocytes enhances NLRP3 signaling and triggers HSC activation and liver fibrosis in young mice in response to liver injury. Unlike young mice, age‐dependent defect of hepatic SIRT1 drives NLRP3 activation and inflammation, which amplifies the initial insult and creates a vicious circle that impairs the capability to resolve fibrosis in old mice even after injury cessation. NLRP3 inhibition by MCC950 ameliorates age‐ and alcohol‐induced liver fibrosis likely by reducing inflammatory cell infiltration and suppressing hepatocyte‐derived danger signaling.

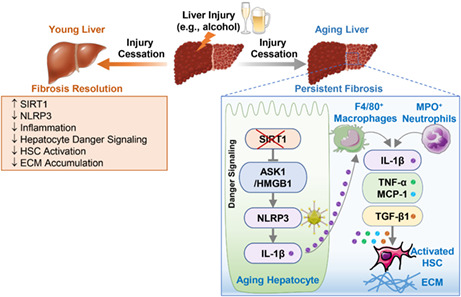

**Abbreviations**
ALD: alcohol‐associated liver disease
ALT: alanine aminotransferase
ASK1: apoptosis signal‐regulating kinase 1
CCl_4_
: carbon tetrachloride
Col1a1: the *gene* that encodes the pro‐alpha1 chains of type I collagen
DAMP: damage‐associated molecular patterns
ECM: extracellular matrix
F4/80: a macrophage marker
FGF21: fibroblast growth factor 21
HMGB1: high mobility group box 1
HSC: hepatic stellate cell
IL‐1β: interleukin‐1β
LOX: Lysyl oxidase
MCC950: a small molecule inhibitor of NLRP3
MMP‐12: metalloproteinase 12
MMPs: metalloproteinases
MCP‐1: monocyte chemoattractant protein‐1
MPO: *myeloperoxidase* that is a neutrophil marker
NLRP3 inflammasome: the NACHT, LRR, and PYD domains‐containing protein 3 inflammasome
PDGFR‐α: platelet‐derived growth factor receptor‐alpha
SIRT1 LKO mice: hepatocyte‐specific SIRT1 knockout mice
SIRT1: a member of the sirtuin family of NAD^+^‐dependent deacetylases
TGF‐β1: transforming growth factor beta 1
TIMP‐1: tissue inhibitor of metalloproteinases
TNF‐α: tumor necrosis factor‐alpha
WT mice: wild‐type mice
α‐SMA: alpha‐smooth muscle actin

## 1 BRIEF

Age‐dependent inhibition of the longevity factor SIRT1 causes induction of the NLRP3 inflammasome and leads to severe and persistent liver fibrosis in old mice even after the cessation of liver injury. SIRT1 deficiency in hepatocytes leads to NLRP3 induction and an inflammatory response that amplifies the initial insult and promotes persistent fibrotic events including activation of hepatic stellate cells and deposition of extracellular matrix proteins. NLRP3 inhibition by MCC950 ameliorates alcohol‐induced liver fibrosis in old mice.

## 2 INTRODUCTION

Aging leads to the progressive impairment of homeostasis at the genomic, cellular, tissue, and, whole organism levels, which increases the risk of chronic liver diseases, such as alcohol‐associated liver disease (ALD) (Pawelec et al., 2014; Ramirez et al., 2017). The aging liver is characterized by multiple cellular events, such as dysregulation of nutrient‐sensing pathways, altered intercellular communication, and genomic instability, which are also described as the hallmarks of aging (Hunt et al., 2019). These changes lead to cellular senescence and low‐grade inflammation, also referred to as inflammaging, which contributes to age‐related phenotypic changes involved in different types of liver cells. Hepatocytes are essential for maintaining metabolic homeostasis; therefore, dysregulation of metabolism in hepatocytes contributes to the susceptibility to age‐related metabolic liver diseases, such as ALD. Age‐related hepatic presinusoidal fibrosis has also been reported (Hunt et al., 2019). However, the impact of age‐related changes in the crosstalk between hepatocytes and hepatic stellate cells (HSCs) during liver fibrosis remains unexplored.

Liver fibrosis is the pathological outcome of a diminished repair response to liver injury (Jun & Lau, 2018; Koyama & Brenner, 2017; Lackner & Tiniakos, 2019). The formation of the fibrotic liver is characterized by HSC activation and excessive accumulation of extracellular matrix (ECM) components, such as collagens. When the liver is damaged, activated HSCs synthesize ECM components and secrete inflammatory mediators; together, these changes initiate the wound‐healing response. When liver damage is minor or non‐repetitive, wound healing is efficient, resulting in only a transient increase in ECM deposition and facilitating the restoration of liver function and architecture. However, when liver injury is severe or repetitive, ECM components continue to accumulate, which in turn results in an inflammatory response, disruption of liver architecture, and ultimately liver failure (Jun & Lau, 2018; Koyama & Brenner, 2017; Lackner & Tiniakos, 2019). Aging‐related liver fibrosis is associated with poor prognosis and even transplants; therefore, treatment in older patients requires different interventions.

Sirtuins have been identified as anti‐aging regulators that mediate the health benefits of calorie restriction and can extend lifespan (Imai & Guarente, 2014). SIRT1 is a member of the sirtuin family of NAD^+^‐dependent deacetylases that control liver metabolism and physiology through production and secretion of FGF21 (Li et al., 2014). SIRT1 is also characterized as a master nutrient sensor that controls lipid homeostasis in hepatocytes through AMPK activation (Hou et al., 2008). Hepatic overexpression of SIRT1 protects against obesity‐induced hepatic ER stress and insulin resistance in young mice (Li et al., 2011). SIRT1 deficiency in hepatocytes promotes the progression of ALD in mice and humans by DEPTOR‐mTORC1 signaling (Chen et al., 2018). However, whether age‐dependent inhibition of hepatic SIRT1 is a driving force to promote liver fibrosis remains elusive.

Alcohol‐associated liver disease (ALD) is one of the leading causes of morbidity and mortality worldwide from chronic liver diseases (Asrani et al., 2019; Crabb et al., 2020). ALD encompasses a spectrum of hepatic manifestations ranging from simple steatosis to steatohepatitis, cirrhosis, and hepatocellular carcinoma (Asrani et al., 2019; Crabb et al., 2020; Lackner & Tiniakos, 2019). Liver inflammation is a significant driver of HSC activation and promotes cirrhosis in humans, which is the 12th leading cause of death in the United States (Asrani et al., 2019; Crabb et al., 2020). The NACHT, LRR, and PYD domains‐containing protein 3 (NLRP3) inflammasome, a unique immune sensor that can be activated by metabolic stressors or damage‐associated molecular patterns (DAMP), triggers sterile inflammation in the absence of overt infection (Gong et al., 2020; Guo et al., 2015). In response to DAMPs, NLRP3 binds to its adaptor protein, apoptosis‐associated speck‐like protein containing a caspase recruitment domain (ASC) and, therefore, activates caspase‐1, which in turn leads to the maturation and secretion of pro‐inflammatory cytokines, such as interleukin‐1β (IL‐1β) (Gong et al., 2020; Guo et al., 2015). However, it is unclear how NLRP3 is regulated in the aging liver and whether this process affects the progression of age‐related liver fibrosis. Here we examine whether NLRP3 inhibition modulates age‐ and alcohol‐induced liver fibrosis by evaluating the efficacy of MCC950, a small‐molecule NLRP3 inhibitor (Coll et al., 2015, 2019).

The present study demonstrates that the age‐dependent decline in SIRT1 is linked to NLRP3 signaling which leads to severe and persistent liver fibrosis during liver injury, even after cessation of injury, in an experimental model of hepatotoxin‐induced liver fibrosis. Strikingly, hepatocyte‐specific SIRT1 ablation caused aberrant HSC activation and severe liver fibrosis in young mice after liver injury, mimicking age‐associated liver fibrosis. Unlike young mice, impaired capacity to resolve established fibrosis in aged mice may depend on disruption of SIRT1 and induction of NLRP3. In an aging mouse model of alcoholic liver fibrosis, NLRP3 inhibition by MCC950 ameliorates chronic‐plus‐binge alcohol feeding‐induced liver fibrosis in old mice. These studies shed light on the mechanism by which the elimination of hepatocyte SIRT1 triggers the NLRP3 inflammatory pathway and impairs the reversibility of liver fibrosis associated with aging.

## 3 EXPERIMENTAL PROCEDURES

### 3.1 Young and old mouse models of CCl_4_ ‐induced liver fibrosis

Mice at 4–6 months of age (young) and mice at 16–20 months of age (old) were intraperitoneally injected with carbon tetrachlorid (CCl_4_) (Cat#319961, Sigma‐Aldrich) diluted 1:4 in olive oil at a dose of 0.5 μL/g body weight twice weekly for 4 weeks with a total of eight injections (Yang et al., 2013). Mice were sacrificed 24 h after the final injection. For the injury recovery phase, the mice were sacrificed 5 days after the final injection, giving time for the mice to recover from CCl_4_‐induced liver injury. All mice were housed in a temperature‐controlled environment with a 12‐h dark/12‐h light cycle. All animal experiments were approved by the Institutional Animal Care and Use Committee (IACUC) at the University of Texas Health Center at San Antonio.

### 3.2 Hepatocyte‐specific SIRT1 knockout (SIRT1 LKO) mice with CCl_4_ ‐induced liver injury

Hepatocyte‐specific SIRT1 knockout (SIRT1 LKO) mice (Alb‐Cre‐positive *SIRT1* floxed mice) were described previously (Chen et al., 2018; Li et al., 2014). We used Alb‐Cre‐negative *SIRT1* floxed mice as age‐matched wild‐type (WT) littermate controls. SIRT1 LKO mice and their WT littermates at 3–5 months of age were intraperitoneally injected with either olive oil or CCl_4_ at a dose of 0.5 μL/g body weight twice weekly for 6 weeks with a total of 12 injections. The mice were sacrificed 24 h after their final injection.

### 3.3 An aging mouse model of alcohol‐induced liver fibrosis and MCC950 treatment

The experimental old mouse model of chronic‐plus‐binge ethanol feeding‐induced liver fibrosis was recently developed, mimicking alcohol drinking patterns and liver fibrosis in patients with ALD (Ramirez et al., 2017; Ren et al., 2022). To determine the effect of MCC950, a selective NLRP3 inhibitor, on the development of alcoholic fibrosis, old mice were treated with MCC950 (Cat#HY‐12815, MedChemExpress) in the Lieber‐DeCarli alcohol liquid diet containing MCC950 (20 mg/kg/day) (Coll et al., 2015, 2019).

### 3.4 Statistical analysis

All statistical analyses were performed using GraphPad Prism 6.0 software (GraphPad Software). If data sets contain two groups, an unpaired two‐tailed Student's t test was employed to determine significant differences. If data sets contain more than two groups, one‐way ANOVA followed by Tukey's post‐hoc test was used to assess significant differences. All data are presented as mean ± standard error of the mean (SEM). For consistency in comparisons, significance in all figures is denoted as follows: *p < 0.05, **p < 0.01, ***p < 0.001, ****p < 0.0001.

## 4 RESULTS

### 4.1 Old mice are more susceptible to liver fibrosis in an experimental model of CCl_4_ ‐induced liver injury

**FIGURE 1:**
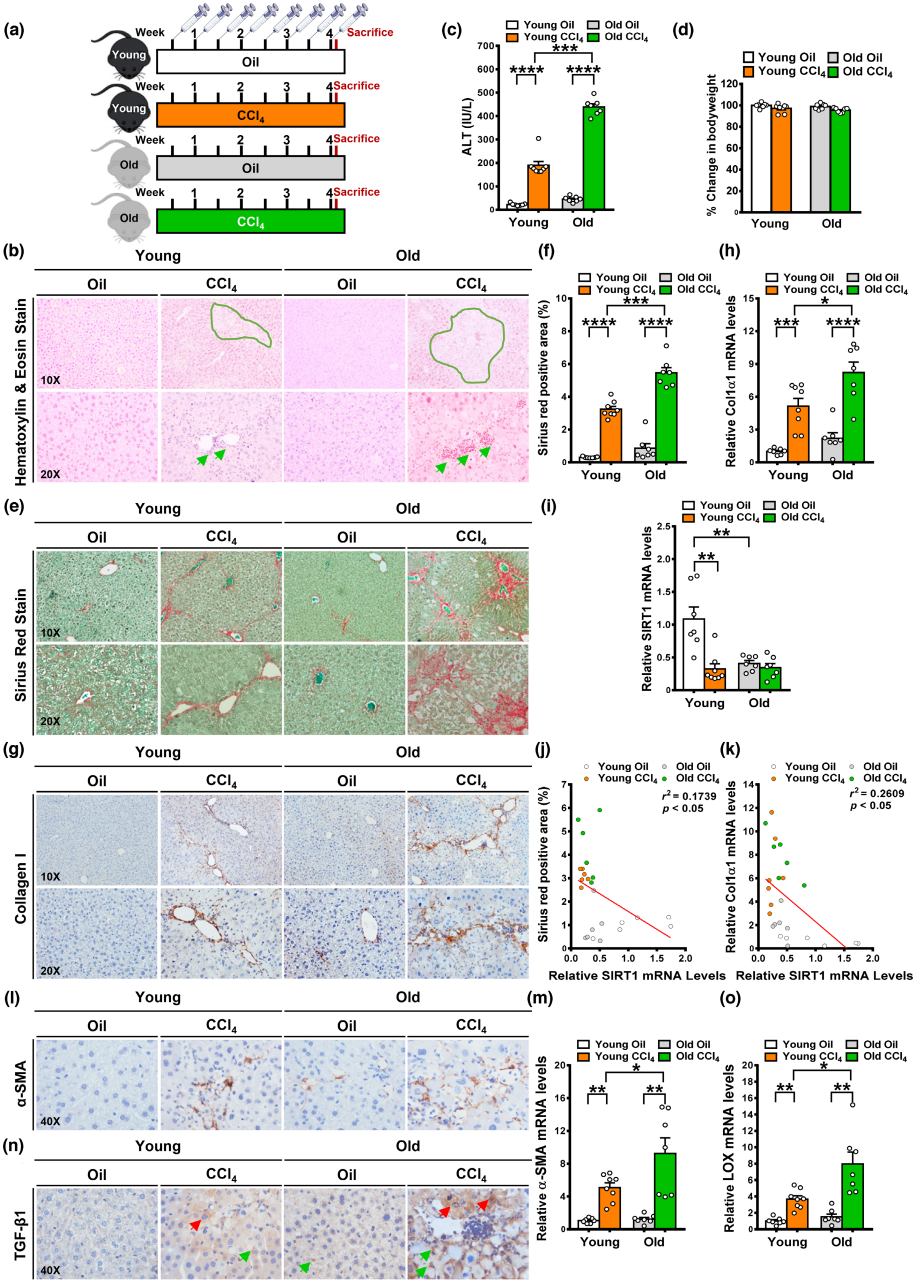
Old mice are susceptible to liver fibrosis in an experimental model of CCl_4_‐induced liver injury. (a) Schematic illustration for a mouse model of CCl_4_‐induced liver fibrosis, which was used to evaluate the reparative response to liver injury in young and old mice. Mice at 4–6 months of age (young) and mice at 16–20 months of age (old) were subjected to injection with either olive oil or CCl_4_ at a dose of 0.5 μL/g body weight twice weekly for 4 weeks. (b) Histopathologic characteristics of hepatocellular necrosis (green outline) and liver inflammation (green arrows) in young and old mice injected with either olive oil or CCl_4_ were assessed by H&E staining. Notably, necrotic hepatocytes with cell swelling, plasma membrane rupture, or loss of the nucleus were seen in CCl_4_‐injected young mice. Extensive necrosis involving larger numbers of necrotic hepatocytes was noted in CCl_4_‐injected old mice. (c) Plasma ALT levels were measured. (d) Assessment of changes in body weight in mice. The change in body weight was expressed as a percentage change between body weight measurements of the mice from the initial and the final day of the experiment. (e) Representative images of Sirius Red staining (collagen is shown as red) revealed the fibrotic structural characteristics in the livers of young and old mice. Notably, collagen fibers were organized in bundles of various thicknesses. Thinner collagen fibrils were seen in young mice, whereas thicker collagen fibers were primarily located in immune cell‐abundant areas of old mice. (f) Positive areas of Sirius Red staining were calculated from five or six random fields of liver sections in each mouse using NIH Image J software. The bar graph represents the percentage of positively stained areas relative to the total tissue areas of liver sections. (g) Immunohistochemistry staining for collagen I revealed a similar pattern of collagen I distribution around the periportal regions in young and old mice, but the intensity of collagen I‐positive staining was increased in old mice in the CCl_4_ model. (h) Hepatic mRNA levels of Col1a1 in mice were analyzed using quantitative reverse‐transcription polymerase chain reaction (qRT‐PCR), normalized to those of GAPDH, and presented as relative levels to oil‐injected young mice. (i) qRT‐PCR analysis of mRNA levels of SIRT1. (j, k) Hepatic SIRT1 levels were negatively correlated with Sirius Red positive areas and Col1a1 levels in young and old mice injected with CCl_4_. (l) Representative immunohistochemistry for α‐SMA, a marker for activated HSCs. (m) Real‐time PCR analysis of mRNA levels of α‐SMA. (n) Representative immunohistochemistry for TGF‐β1. Notably, positive staining for TGF‐β1 was visualized in hepatocytes (red arrows) and other cell types (green arrows) in fibrotic livers. (o) Real‐time PCR analysis showed that the induction of LOX was much higher in old mice than that in young mice after repeated liver injury. All images were acquired using 10×, 20×, or 40× objectives. The data were expressed as means ± SEM, n = 7–8. *p < 0.05, **p < 0.01, ***p < 0.001, or ****p < 0.0001 between two groups.

To initially assess liver injury in young and old mice, H&E staining was performed, and plasma alanine aminotransferase (ALT) levels were measured. Rupture of the plasma membrane results in the release of cellular constituents into the extracellular environment, a pathological process that elicits an inflammatory response (Weinlich et al., 2017). As shown in Figure 1a–d, H&E staining showed that after repeated injections of CCl_4_, hepatocyte necrosis in the periportal regions of young and old mice was characterized by cytoplasmic swelling, cell rupture, and loss of the nucleus; an increase in necrotic areas was more pronounced in older mice. Notably, more inflammatory foci composed predominantly of polymorphonuclear cells around areas of cell death were also observed in old mice. Furthermore, plasma ALT levels were approximately twofold higher in old mice than in young mice. Alterations of body weight were comparable among four groups of young and old mice. Taken together, these results suggest that aging increases the susceptibility to liver injury and triggers a highly inflammatory response.

Liver fibrosis is characterized by accumulation of ECM proteins, which disrupt the liver microenvironment (Koyama & Brenner, 2017). Liver fibrosis was assessed by Sirius Red staining and quantified by measuring positively stained areas. As shown in Figure 1e–h, in olive oil‐injected controls, Sirius Red staining and quantification showed a pattern of perisinusoidal fibrosis and an approximately twofold increase in the positively stained areas of old mice, with minimal positive staining seen in young mice. Immunohistochemistry for collagen I further confirmed the perisinusoidal fibrosis in the livers of oil‐injected old mice. Immunohistochemistry staining for alpha‐smooth muscle actin (α‐SMA) also showed an elevated number of activated HSCs, the cell type that is the key source of ECM compounds, in the normal aging liver (Figure 1l). Notably, there was a modest but statistically insignificant increase in mRNA levels of fibrogenic markers, Col1a1, the gene that encodes the pro‐alpha1 chains of type I collagen and α‐SMA (Figure 1h,m), possibly because the ECM deposition in the normal aging liver reflects the imbalance of hepatic fibrogenesis and fibrolysis. After repeated injections of CCl_4_, the degree of bridge fibrosis was more severe in older mice than that in younger mice; amounts and staining intensity of α‐SMA^+^ HSC cells were also higher in older mice (Figure 1e–g). Similarly, mRNA levels of Col1a1 and α‐SMA were nearly doubled in old mice (Figure 1h,m). Thus, old mice are prone to developing perisinusoidal fibrosis with HSC activation and exhibit greater susceptibility to liver fibrosis during chronic liver injury.

### 4.2 Aging exacerbates liver fibrosis by hampering SIRT1 and dysregulating extracellular matrix remodeling

To elucidate the possible mechanism underlying age‐related liver fibrosis, we found that hepatic levels of the longevity factor SIRT1 declined dramatically by ~70% with age (Figure 1i), mimicking the inhibitory effect of CCl_4_ administration on SIRT1. Notably, the mRNA levels of SIRT1 were negatively correlated Sirius Red positive areas and Col1a1 levels in mice with or without CCl_4_ administration (Figure 1j,k). Interestingly, positive staining for transforming growth factor beta 1 (TGF‐β1), the profibrogenic cytokine, was present in hepatocytes and non‐parenchymal cells, such as immune cells, in both young and old mice injected with CCl_4_. However, this induction was further potentiated in old mice. Lysyl oxidase (LOX) converts lysine molecules into highly reactive aldehydes that form the cross‐links of ECM proteins and promote cross‐linking and stabilization of collagens during liver fibrosis (Cox et al., 2013). We found that CCl_4_ administration resulted in a 3.6‐fold increase in LOX expression in young mice, and induction of LOX was ~twofold higher in old mice (Figure 1o). This suggests that covalent cross‐linking of collagen fibrils induced by LOX is involved in the development of a stabilized ECM in the aging liver, leading to less ECM degradation and the formation of dense collagen fibers. Collectively, progressive liver fibrosis during aging is likely attributed to pronounced suppression of SIRT1, induction of TGF‐β1‐sensitized HSCs, and aberrant ECM remodeling.

### 4.3 Hepatocyte‐specific SIRT1 deficiency mimics the ability of aging to exacerbate liver fibrosis in mice

**FIGURE 2:**
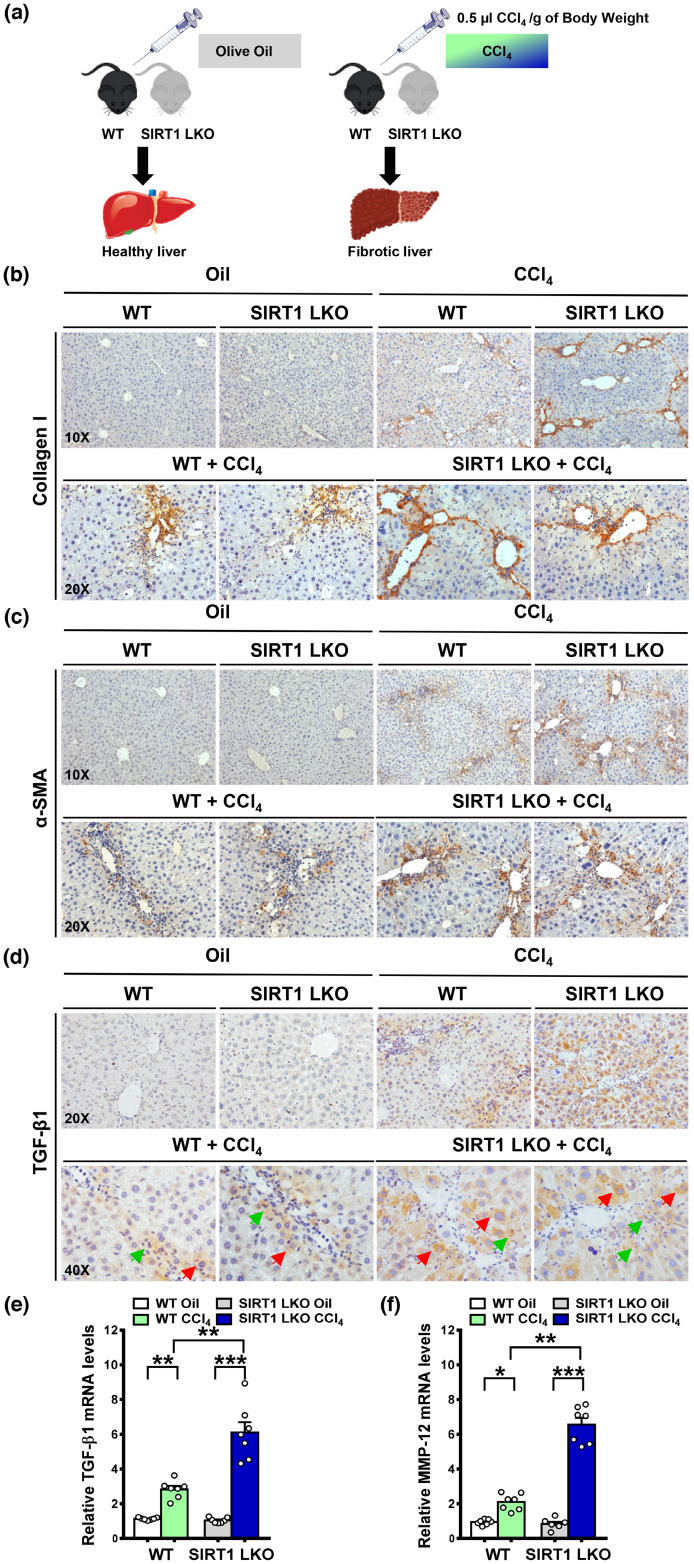
Deletion of SIRT1 in hepatocytes increases the susceptibility of young mice to CCl_4_‐induced liver fibrosis. (a) Wild‐type (WT) and SIRT1 LKO mice at 3–5 months of age were subjected to injection with either olive oil or CCl_4_ at a dose of 0.5 μL/g body weight twice weekly for 6 weeks. (b) Representative immunohistochemistry for collagen I. A similar pattern for collagen I positive staining was present primarily around the periportal regions in WT mice in the CCl_4_ model, but higher collagen I deposition was noted in SIRT1 LKO mice. (c) Representative immunohistochemistry for α‐SMA. Intense staining for α‐SMA was detected in fibrotic areas of WT mice; the distribution and intensity of positive staining for α‐SMA were higher in SIRT1 LKO mice. (d) Representative immunohistochemistry for TGF‐β1. Immunostaining signals for TGF‐β1 were located primarily in hepatocytes (red arrows) and other cell types (green arrows). (e, f) Real‐time PCR analysis of mRNA levels of TGF‐β1 and MMP‐12. All images were acquired using 10×, 20×, or 40× objectives. The data were expressed as means ± SEM, n = 6–8. *p < 0.05, **p < 0.01, or ***p < 0.001 between two groups.

Given the evidence that inhibition of SIRT1 is linked to excessive ECM deposition in old mice within the context of liver injury (Figure 1), we sought to determine the role of hepatocyte SIRT1 loss in liver fibrosis by using hepatocyte‐specific SIRT1 knockout (SIRT1 LKO, AlbCre^+^SIRT1^f/f^) mice (Li et al., 2014). As shown in Figure 2a–d, immunohistochemistry revealed that collagen I deposition was increased in WT mice after repetitive administrations of CCl_4_, compared with minimal staining in age‐matched oil controls. Interestingly, hepatocyte SIRT1 inactivation led to the development of more severe fibrosis with extensive collagen deposition. To delineate the mechanism contributing to the profibrotic effect of hepatocyte SIRT1 ablation, we found that the α‐SMA^+^ HSCs were highly distributed around periportal areas and fibrotic bands in both WT and SIRT1 LKO mice following CCl_4_ administrations; amounts of α‐SMA^+^ HSCs were further increased in the SIRT1 LKO mice, consistent with excessive ECM accumulation. Furthermore, the expression of TGF‐β1 was significantly upregulated in hepatocytes and other cell types of SIRT1 LKO mice. Liver fibrosis is a dynamic process resulting from an imbalance between ECM production (fibrogenesis) and degradation (fibrolysis). Metalloproteinases (MMPs) are zinc‐dependent endopeptidases that are involved in fibrosis progression through their ability to degrade ECM components (Koyama & Brenner, 2017). To test the possibility that dynamic matrix remodeling plays a role in pathological ECM degradation in SIRT1 LKO mice, we observed a twofold increase in the matrix remodeling enzyme—metalloproteinase 12 (MMP‐12)—in WT mice upon CCl_4_ injection, but the induction of MMP12 was further enhanced by threefold in the SIRT1 LKO mice (Figure 2e,f), suggesting that while a fibrogenic process can account for the severity of fibrosis observed in SIRT1 LKO mice, dysregulation of fibrolysis may also contribute to the progression of liver fibrosis. Collectively, loss of hepatocyte‐specific SIRT1 may represent a novel mechanism that controls the crosstalk between hepatocytes and HSCs through hepatocyte‐derived cytokines such as TGF‐β1, leading to hyperactivation of HSCs and disruption of ECM homeostasis.

### 4.4 Young mice are able to reverse liver fibrosis after cessation of injury by inhibiting hepatic stellate cell activation

Accumulated evidence indicates that certain circumstances permit the resolution of fibrosis when the underlying causes of injury are eradicated (Jun & Lau, 2018). We focused on studying the mechanism responsible for fibrosis regression as a means for developing new therapeutic approaches relevant to human disease. To evaluate dynamic changes in HSC activation and reversal of fibrosis, liver tissues were harvested in young mice at baseline, 24 h post‐injections, and after 5 days of recovery. As shown in Figure S1A–H, plasma ALT levels were increased more than sevenfold by CCl_4_ injection; this elevation was reduced by ~70% after a 5‐day recovery, suggesting a rapid recovery of liver injury in young mice. H&E and Sirius Red staining showed hepatocellular necrosis as well as accumulation of ECM components and bridging fibrosis in young mice within 24 h of the last CCl_4_ injection, which rapidly decreased 5 days after discontinuation of CCl_4_. Consistently, mRNA levels of Col1a1 were increased ~fivefold by CCl_4_ administration in young mice compared with oil controls. In contrast, this induction was reduced by nearly 40% during the recovery period. To examine the relative contribution of HSCs to the regression of fibrosis in young mice, we found that the number of α‐SMA^+^ HSCs was increased by repeated CCl_4_ administration, but clearance of activated HSCs was evident upon cessation of further injury, consistent with collagen I disappearance. Similarly, expression of α‐SMA was increased by ~eightfold upon CCl_4_ injections but reduced by ~50% during the recovery, suggesting that fibrosis regression occurs in young mice predominantly through the clearance of activated HSCs.

### 4.5 Fibrosis regression in young mice is associated with SIRT1 restoration and inflammation reduction following the cessation of liver insult

The above findings indicate that SIRT1 ablation accelerates fibrosis progression in young mice, thus we asked whether hepatic SIRT1 might be altered in mice following cessation of injury. As shown in Figure S1I–K, SIRT1 levels were markedly decreased by ~70% during liver injury but were increased by approximately twofold in the absence of further injury, suggesting that restoration of SIRT1 may represent a protective mechanism against fibrosis in young mice. Interestingly, TGF‐β1^+^ cells were lowered after a 5‐day recovery, consistent with the clearance of HSCs. Moreover, expression of the ECM regulators, MMP‐9, TIMP‐1, and LOX, was significantly increased by three‐ to fivefold in mice after liver injury and decreased after fibrosis regression, reaching nearly the baseline level seen in oil‐injected controls. Therefore, fibrosis regression in young mice is possibly mediated by restoration of SIRT1, ECM homeostasis, and inhibition of the TGF‐β1‐dependent fibrogenesis. Liver inflammation was histologically assessed by immunohistochemistry for immune cell markers, F4/80 and MPO, and quantified by real‐time PCR analysis for mRNA levels of pro‐inflammatory markers and cytokines. As shown in Figure S1L,M, positive staining for F4/80, a macrophage marker, was increased in the portal triad region of young mice within the context of CCl_4_ injection. Accumulation of cells positively stained for MPO, a neutrophil marker, was also observed in the perivascular region. Conversely, recruitment of enlarged F4/80^+^ macrophages and MPO^+^ neutrophils was significantly reduced after injury recovery. Expression of pro‐inflammatory cytokines and chemokines, such as tumor necrosis factor‐alpha (TNF‐α), IL‐1β, and monocyte chemoattractant protein‐1 (MCP‐1) (also known as CCL2), was reduced by 55%–72% in young mice upon injury recovery. Collectively, the reversibility of fibrosis in young mice may be, at least in part, mediated by increasing SIRT1 signaling and decreasing infiltration of inflammatory cells.

### 4.6 Aging leads to an impaired capacity to resolve established fibrosis even after cessation of liver injury

**FIGURE 3:**
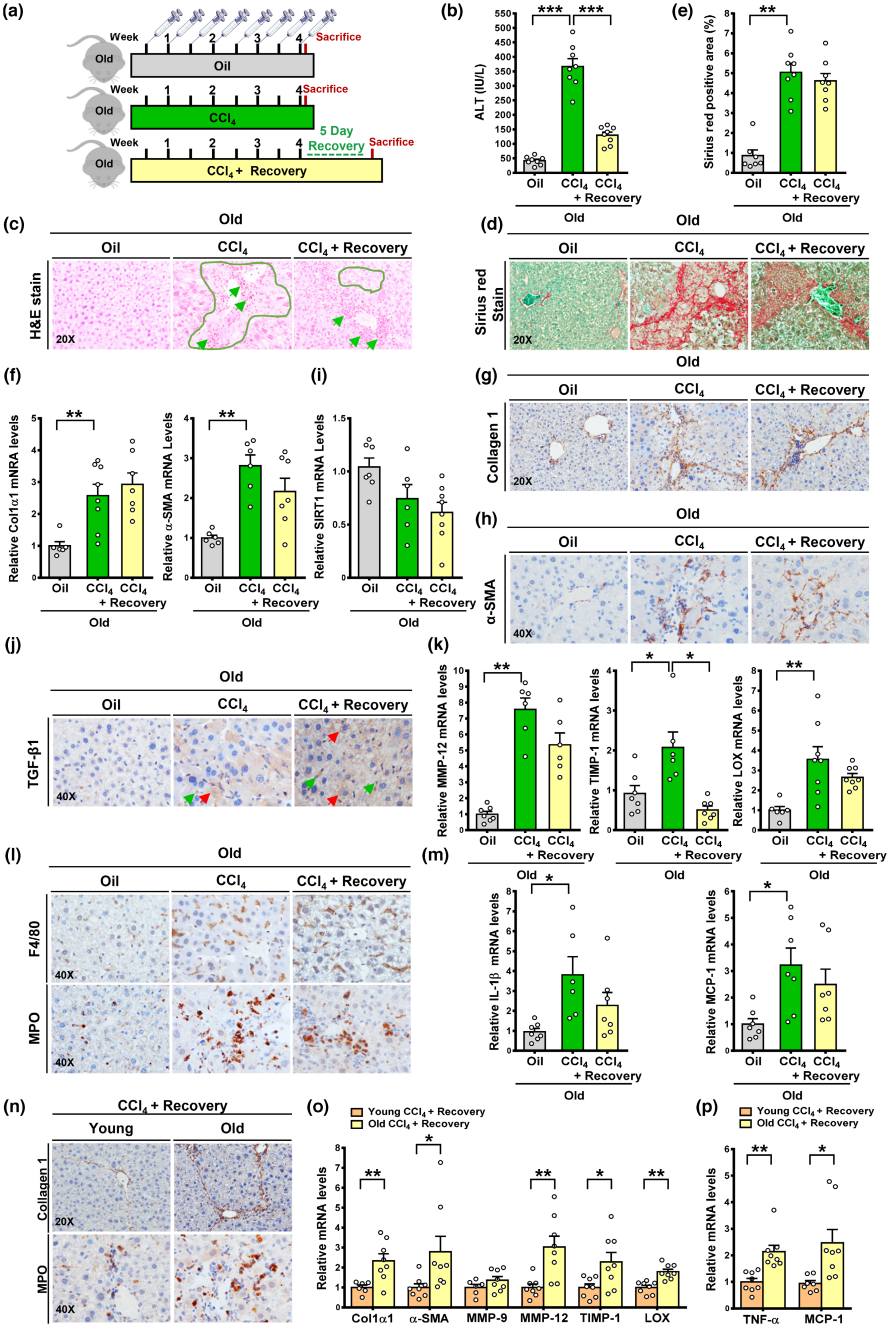
Persistent liver fibrosis in old mice after cessation of liver injury is associated with inflammation induction. (a) Schematic illustration of the time course of both liver fibrosis and resolution of established fibrosis in old mice in the CCl_4_ model. (b) Assessment of plasma ALT levels. (c) Representative H&E staining of liver sections in oil‐injected old mice (Oil), in old mice 24 h post‐injection (CCl_4_), and in old mice 5 days after the last CCl_4_ injection (CCl_4_ + Recovery). (d, e) Sirius Red staining and quantification showed that aging increased the susceptibility of mice to CCl_4_‐induced liver fibrosis even after cessation of liver injury. (f) Real‐time PCR analysis showed that mRNA levels of Col1a1 and α‐SMA were increased by CCl_4_ injections, which were not significantly altered upon cessation of CCl_4_ administration. (g) Immunohistochemistry for collagen I showed a similar distribution and intensity for collagen deposition in old mice between 24 h post‐injection and 5 days after cessation of injury. (h) Immunohistochemistry for α‐SMA showed that elevated α‐SMA^+^ HSCs were primarily distributed around portal areas and fibrotic bands in old mice during liver injury and after cessation of injury. (i) Real‐time PCR analysis of hepatic expression of SIRT1. (j) Immunohistochemistry revealed positive staining for TGF‐β1 in hepatocytes (red arrows) and other cells (green arrows) in old mice. (k) Real‐time PCR analysis of ECM regulators including MMP‐12, TIMP1, and LOX. (l) Immunohistochemistry for F4/80^+^ macrophages and MPO^+^ neutrophils. (m) Real‐time PCR analysis showed that the expression of IL‐1β and MCP‐1 was increased in old mice that underwent CCl_4_ injections and persisted upon cessation of CCl_4_ administration. (n) Immunohistochemistry for collagen I and MPO. (o) Real‐time PCR analysis of fibrosis markers, including Col1a1, a‐SMA, MMP‐9, MMP‐12, TIMP‐1, and LOX. (p) Real‐time PCR analysis showed that upon cessation of CCl_4_ administration, expression of inflammation regulators, including TNF‐α and MCP‐1, were upregulated in old mice. All images were acquired using 20× or 40× objectives. The data were expressed as means ± SEM, n = 6–8. *p < 0.05, **p < 0.01, or ***p < 0.001, between two groups.

To determine the effect of aging on resolution of established fibrosis, the fibrotic response was characterized in old mice in the CCl_4_ model at baseline, 24 h post‐injury, and after 5 days of recovery (Figure 3a). Plasma ALT levels were increased more than ninefold in CCl_4_‐injected old mice and reduced by ~60% after the recovery period (Figure 3b), which were higher than those seen in young mice (Figure S1B). H&E and Sirius Red staining showed comparable severity of hepatocellular necrosis, liver inflammatory foci, and bridge fibrosis in old mice at 24 h post‐injury and after the 5‐day recovery (Figure 3c–e). In contrast to the fibrosis resolution which occurred in young mice (Figure S1), mRNA levels of fibrogenic markers, including Col1a1 and a‐SMA, were increased by over twofold in old mice following CCl_4_ administration, and this elevation persisted following the cessation of injury (Figure 3f). Immunohistochemistry for collagen 1 and α‐SMA further confirmed perisinusoidal fibrosis in the livers of animals receiving oil injections; aging delayed regression of fibrosis and elimination of activated HSCs during the recovery period (Figure 3g,h). As illustrated in Figure 3i–k, upon the cessation of injury, a decrease in SIRT1 was observed but was not found to be statistically significant, probably due to the low basal level of SIRT1 in old mice. Moreover, TGF‐β1^+^ cells were elevated in old mice upon CCl_4_ injections and remained persistent upon discontinued injury, parallel with the accumulation of a‐SMA^+^ HSCs. Although TIMP‐1 levels were reduced, a sustained increase in MMP‐12 and LOX was seen, highlighting that cross‐linking of ECM proteins by LOX may account for the accumulation of thicker fibrotic septa and confer resistance to fibrolysis. Collectively, prolonged inhibition of SIRT1 contributes to the diminished capacity for the regression of abnormal ECM remodeling and established fibrosis during aging.

### 4.7 Persistent liver fibrosis in old mice is associated with inflammatory cell infiltration

We sought to determine the effect of injury cessation on inflammation in old mice. As shown in Figure 3l,m, after CCl_4_ administration, recruitment of enlarged F4/80^+^ macrophages and MPO^+^ neutrophils, as well as expression of IL‐1β and MCP‐1 were dramatically increased more than threefold in old mice. Following the cessation of injury, infiltrated macrophages and neutrophils, as well as the rise in IL‐1β and MCP‐1 levels, were not significantly altered in old mice upon the cessation of injury, suggesting that enhanced inflammatory cell expansion may explain persistent fibrotic scars during aging. To further compare a fibrotic phenotype between the young and old mice during the regression phase, we found that the older mice exhibited more severe fibrosis after the cessation of injury (Figure 3n). Similarly, the mRNA levels of fibrogenic markers, Col1a1 and a‐SMA, were elevated by over twofold in old mice. Expression of ECM regulators, including MMP‐12, TIMP‐1, and LOX, was much higher in old mice than that in young mice (Figure 3o), suggesting that the formation of a mature and stabilized ECM is a possible factor contributing to limited fibrosis reversibility in the aging liver. Moreover, intrahepatic infiltration of MPO^+^ cells as well as expression of TNF‐α and MCP‐1 were increased by over twofold in older mice compared with younger mice (Figure 3p). Suppression of hepatic SIRT1, coupled with infiltration of inflammatory cells, could explain the inability to resolve established fibrosis during aging.

### 4.8 Hepatocyte SIRT1 ablation enhances NLRP3 inflammasome signaling in young mice after liver injury

**FIGURE 4:**
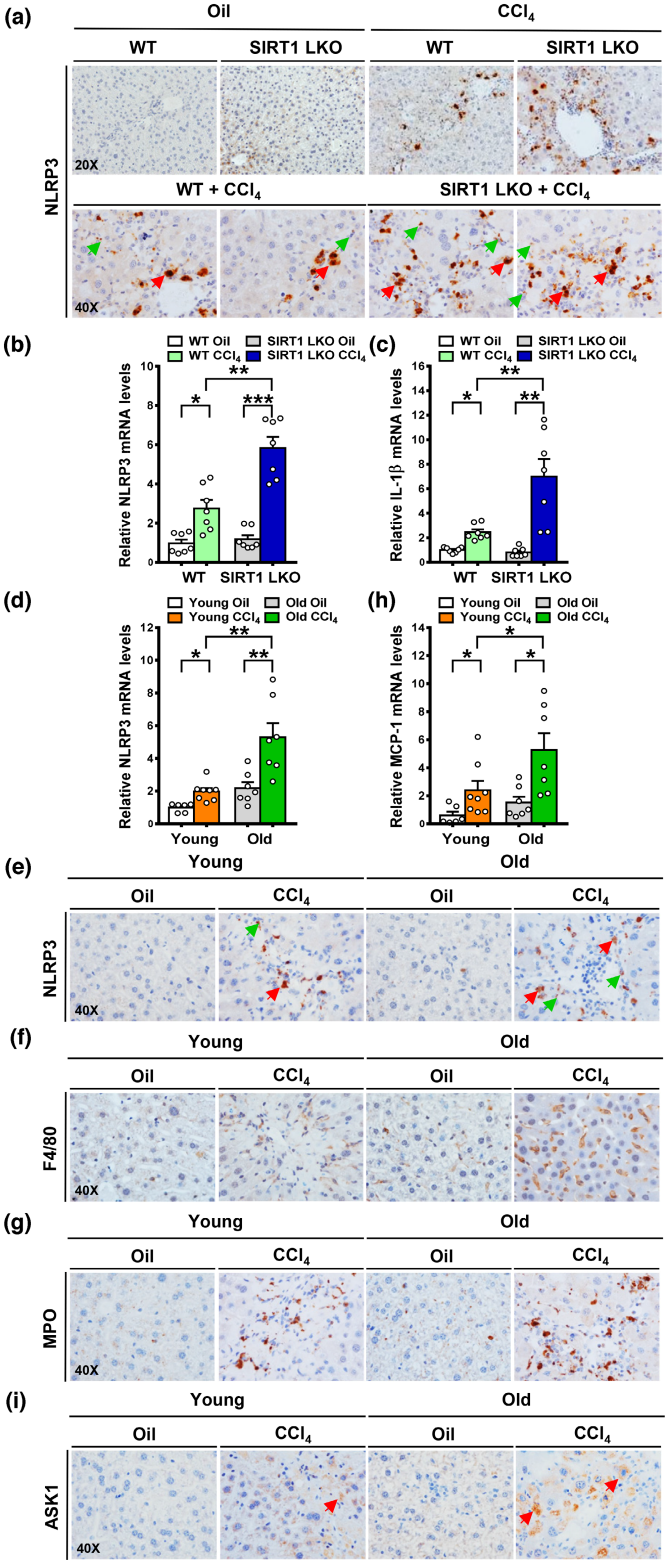
SIRT1 deficiency in hepatocytes enhances NLRP3 signaling and mimics the effect of aging on NLRP3‐mediated inflammation. (a) Immunohistochemistry showed that cells positively stained for NLRP3 were mainly located in damaged hepatocytes with nuclear degradation (red arrows) and inflammatory cells (green arrows) in young WT mice after CCl_4_ administration, and this elevation was further enhanced in young SIRT1 LKO mice. (b, c) Real‐time PCR analysis showed that mRNA levels of NLRP3 and IL‐1β were increased in WT mice, and this induction was further potentiated in SIRT1 LKO mice. (d) Real‐time PCR analysis showed that mRNA levels of NLRP3 were significantly increased in young and old mice after CCl_4_ administration, and the induction of NLRP3 was higher in older mice than in younger mice. (e) Immunohistochemistry showed that cells positively stained for NLRP3 were visualized in damaged hepatocytes with nuclear degradation (red arrows) and inflammatory cells (green arrows). (f, g) Immunohistochemistry for F4/80, a macrophage marker, and MPO, a neutrophil marker. (h) Real‐time PCR analysis for mRNA levels of MCP‐1. (i) Immunohistochemistry revealed positive staining for ASK1 in damaged hepatocytes (red arrows). All images were acquired using 20×, or 40× objectives. The data were expressed as means ± SEM, n = 6–8. *p < 0.05 and **p < 0.01 or ***p < 0.001 between two groups.

Having demonstrated that SIRT1 LKO mice develop more severe fibrosis in the CCl_4_ model (Figure 2), we further characterized the potential role of hepatocyte SIRT1 in regulating NLRP3 signaling and its functional consequences. As shown in Figure 4a–c, after liver injury, NLRP3 appeared to be activated in dying hepatocytes that exhibited cytoplasmic swelling. While NLRP3^+^ cells were rarely seen in oil controls, a high number of hepatocytes and other cell types that were positively stained for NLRP3 were detected in WT mice after CCl_4_ injection. The number of NLRP3^+^ cells was much higher in SIRT1 LKO mice, suggesting that SIRT1 deficiency leads to NLRP3 activation in damaged hepatocytes. Accordingly, mRNA levels of NLRP3 were increased by ~threefold and ~sixfold in WT mice and SIRT1 LKO mice, respectively. Consequently, the expression of the cytokine IL‐1β, a downstream effector of NLRP3, was significantly upregulated by ~twofold in WT mice. The induction of IL‐1β was further amplified by threefold in SIRT1 LKO mice, suggesting the presence of NLRP3^+^ cells and elevation of IL‐1β as key pathological features of liver fibrosis associated with SIRT1 loss.

### 4.9 Age‐dependent decline of SIRT1 promotes the development of liver fibrosis at least in part through enhancing NLRP3 signaling

Our above data suggest that inflammaging is implicated in persistent fibrosis in old mice (Figure 3). To understand the mechanism that mediates the distinct inflammatory reactions in young and old mice, we asked whether the NLRP3 inflammasome changes with age. As shown in Figure 4d–g, real‐time PCR analysis revealed that old mice receiving oil injection had a tendency toward higher basal levels of NLRP3 and MCP‐1 compared with young mice, although this increase was not statistically significant. Strikingly, mRNA levels of NLRP3 were significantly elevated by ~twofold by CCl_4_ injection in young mice, but the induction of NLRP3 was more pronounced in older mice, consistent with fibrosis severity (Figure 3d). A significant number of NLRP3^+^ cells, as well as enlarged F4/80^+^ macrophages and MPO^+^ neutrophils, were observed in young and old mice injected with CCl_4_, but old mice displayed higher number and intensity. Expression of MCP‐1 was increased by ~threefold and ~fivefold in the young and old mice, respectively. These results uncover that age‐related SIRT1 loss drives activation of NLRP3 and inflammation in response to liver injury. Emerging evidence indicates that apoptosis signal‐regulating kinase 1 (ASK1), the kinase that is highly expressed in hepatocytes and is activated by various stressors such as ER stress, is required for NLRP3 activation (Place et al., 2018). To test the hypothesis that aberrant activation of NLRP3 might be relevant to ASK1 signaling in CCl_4_‐injected old mice, we found that upon CCl_4_ injection, ASK1‐positive staining was present in hepatocytes with nuclear degradation, indicative of damaged hepatocytes. A higher number of ASK1^+^ hepatocytes was noted in older mice than in younger mice (Figure 4i), consistent with the elevation of NLRP3^+^ cells. These results suggest that activation of ASK1 stress signaling may be responsible for induction of NLRP3 in damaged hepatocytes of CCl_4_‐injected old mice.

### 4.10 Aging results in an impaired ability to resolve established fibrosis likely through NLRP3 induction

**FIGURE 5:**
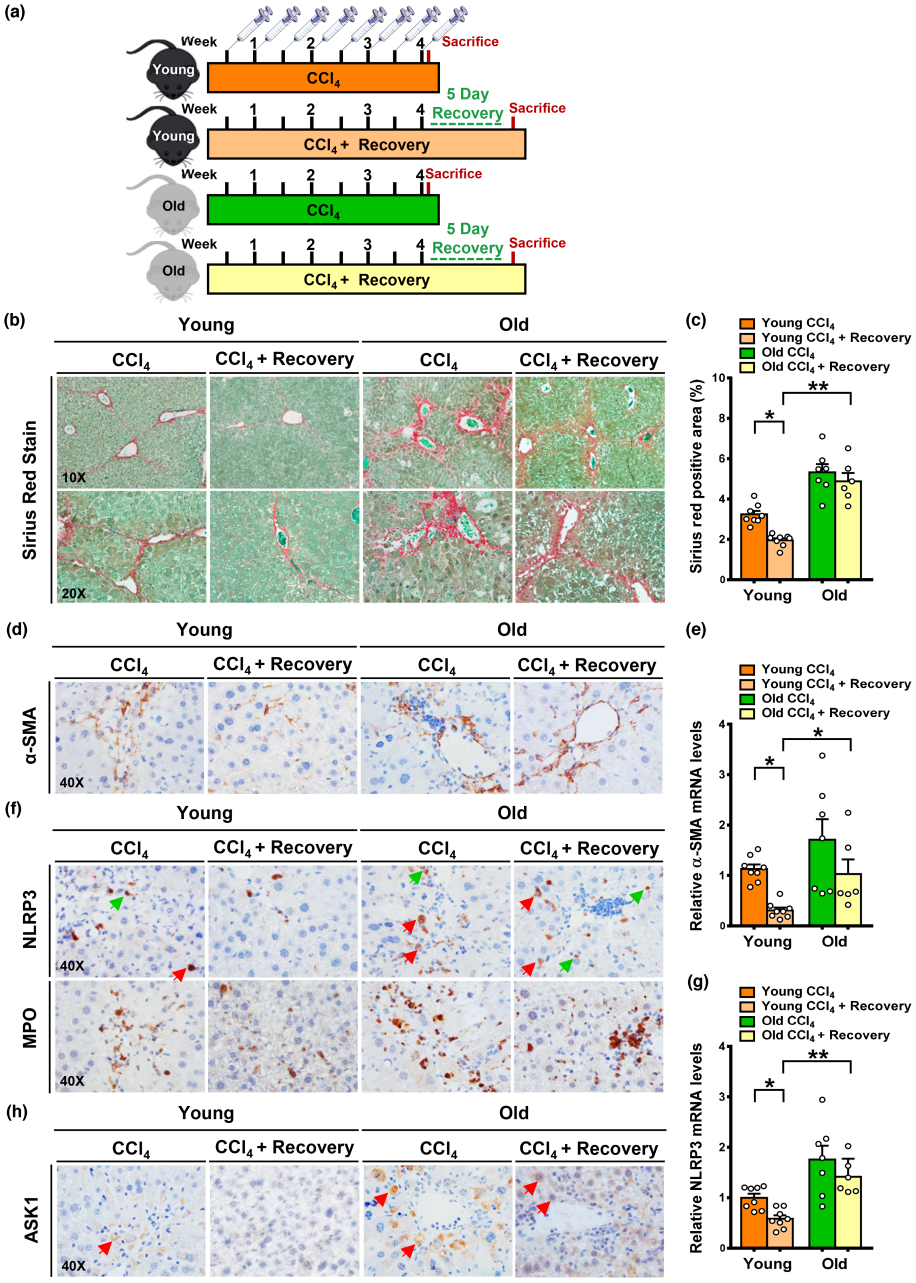
NLRP3‐mediated inflammation contributes to the pathogenesis of age‐associated persistent fibrosis even after cessation of liver injury. (a) Schematic illustration of two distinct phases of liver injury and cessation of injury in young and old mice in the CCl_4_ mouse model. (b, c) Sirius Red staining and quantification showed that after the recovery period, fibrosis regression occurred in young mice, whereas severe fibrosis around the periportal regions was still seen in old mice. (d) Immunohistochemistry showed that the number of α‐SMA^+^ HSCs was significantly higher in older mice during liver injury. After injury recovery, clearance in α‐SMA^+^ HSCs was evident in young mice, whereas accumulation of α‐SMA^+^ HSCs was sustained in older mice. (e) Real‐time PCR analysis showed the induction of α‐SMA expression caused by CCl_4_ administration was eliminated in young mice after the injury recovery, but the expression of α‐SMA was not significantly altered in old mice. (f) Immunohistochemistry for NLRP3 and MPO showed that after CCl_4_ injection, NLRP3^+^ hepatocytes (red arrows) and NLRP3^+^other cell types (green arrows), as well as MPO^+^ neutrophils were higher in old mice than in young mice. After 5 days of recovery, these positively stained cells were reduced in young mice, but not in old mice. (g) Real‐time PCR analysis of NLRP3. (h) Immunohistochemistry showed that following CCl_4_ injection, positively stained cells for ASK1 were detected in damaged hepatocytes with nuclear degradation (red arrows) in both young and old mice, but ASK1^+^ hepatocytes were higher in old mice. After the recovery period, ASK1^+^ hepatocytes were reduced in young mice, whereas a high number of ASK1^+^ hepatocytes were still present in old mice. All images were acquired using 10×, 20×, or 40× objectives. The data were expressed as means ± SEM, n = 6–8. *p < 0.05, or **p < 0.01 between two groups.

As depicted in Figure 5a–e, Sirius Red staining and quantification revealed that hepatic fibrosis was dramatically increased in the periportal regions of old mice subjected to CCl_4_ injection and persisted even after the recovery period. Conversely, young mice were less predisposed to fibrosis after liver injury and recovered better. Similarly, the accumulation of α‐SMA^+^ HSCs appeared indistinguishable upon CCl_4_ injection and after cessation of injury, whereas clearance of α‐SMA^+^ HSCs was remarkably increased in young mice during the regression phase. Accordingly, expression of α‐SMA was quantitatively higher in old mice than in young mice and functionally, activation of HSCs contributes to persistent fibrosis in old mice. Collectively, HSC clearance and fibrosis resolution occur in young mice, whereas persistent fibrosis with the accumulation of HSCs was observed in old mice after cessation of injury. As shown in Figure 5f,g, following CCl_4_ administration, NLRP3^+^ cells were detected in the two age groups, but the number of NLRP3^+^ cells was substantially higher in older fibrotic livers. Specifically, following cessation of liver injury, NLRP3^+^ cells were absent in young mice, whereas the number of NLRP3+ cells remained consistent in old mice. Accordingly, elevation of NLRP3 in fibrotic livers was downregulated by ~50% in young mice, but the expression of NLRP3 remained elevated in old mice. Consistent with the upregulation of IL‐1β in age‐related persistent fibrosis (Figure 3m), NLRP3 expression was further potentiated by ~twofold in old mice. The inflammatory response, as reflected by MPO^+^ cell infiltration, was transient in young mice, whereas accumulation of MPO+ cells remained markedly persistent in aged mice, consistent with pronounced induction of NLRP3. As shown in Figure 5h, the number of ASK1^+^ hepatocytes was increased in young mice after CCl_4_ injection but was largely blunted after injury recovery. These results suggest that young mice can decrease NLRP3 signaling possibly through inhibition of hepatocyte‐derived stress signaling, such as ASK1. In contrast, older mice still displayed an accumulation of ASK1^+^ cells, in conjunction with hyperactivation of NLRP3. Collectively, the inability to resolve established fibrosis in the liver of old mice may be associated with the acquisition of an NLRP3‐induced inflammatory phenotype, which is mediated, at least in part, by ASK1 signaling in hepatocytes.

### 4.11 Inhibition of the NLRP3 inflammasome by MCC950 ameliorates hepatic inflammation in old mice after chronic‐plus‐binge alcohol feeding

**FIGURE 6:**
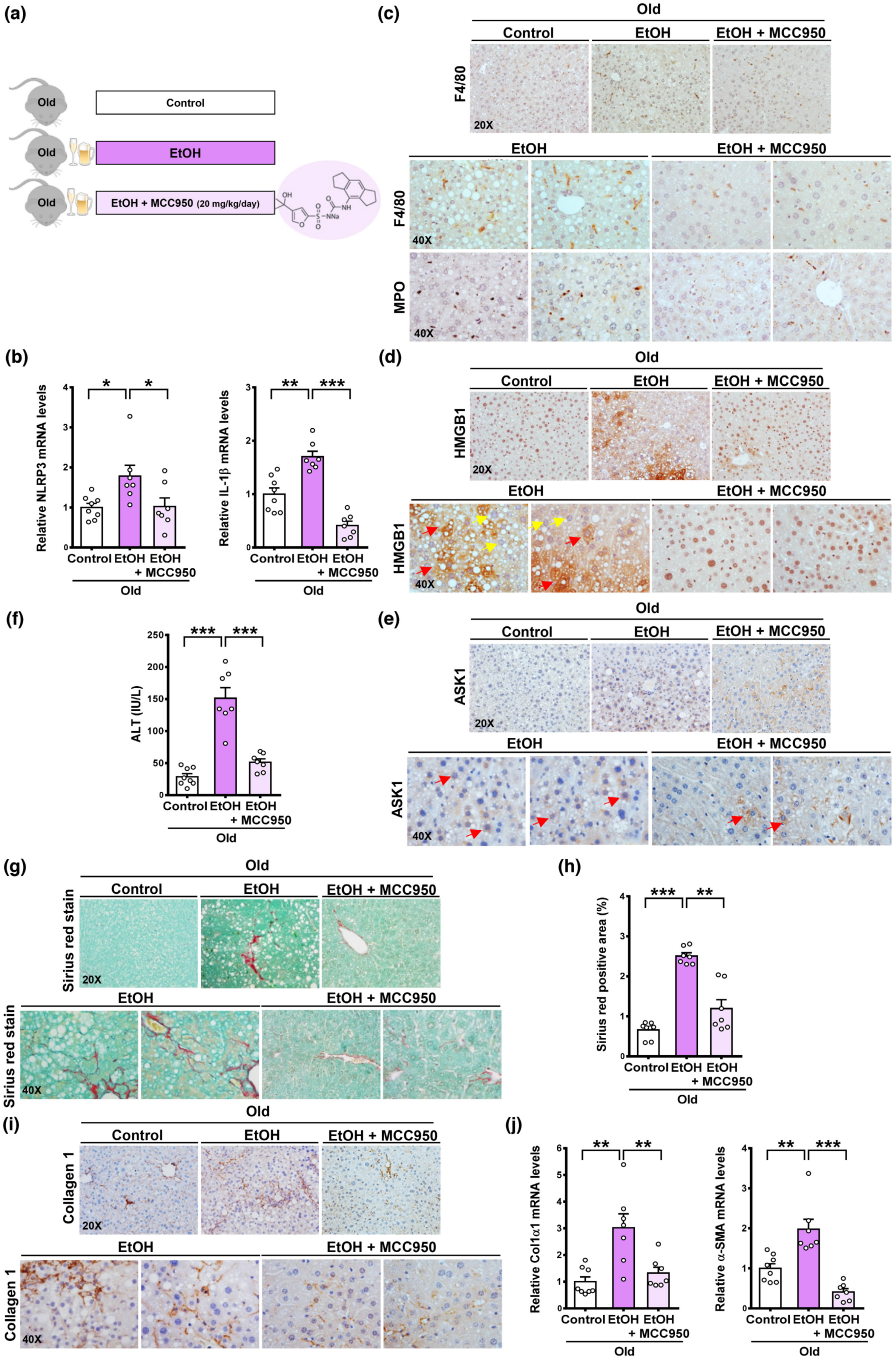
Inhibition of the NLRP3 inflammasome by MCC950 ameliorates age‐ and alcohol‐induced liver inflammation and fibrosis in mice. (a) Schematic illustration of an old mouse model of chronic‐plus‐binge alcohol feeding‐induced liver fibrosis and the design of MCC950 treatment (20 mg/kg/day), starting from the first day of the ethanol diet. (b) Real‐time PCR analysis showed that expression of NLRP3 and IL‐1β was markedly upregulated in old mice after chronic‐plus‐binge alcohol feeding but was significantly diminished by MCC950 treatment. (c) Immunohistochemistry for F4/80^+^ macrophages and MPO^+^ neutrophils. (d) Immunohistochemistry showed that positive staining for HMGB1 was primarily visualized in the nuclei of hepatocytes in control mice. Upregulation of HMGB1 and its translocation from the nucleus to the cytoplasm were observed in hepatocytes from old mice after chronic‐plus‐binge ethanol feeding. Positive staining for HMGB1 in both the cytoplasm and nucleus (red arrows) was detected in some hepatocytes, and nuclear export of HMGB1 (yellow arrows) was also found in other hepatocytes, indicating different stages of the cytoplasmic translocation of HMGB1. In particular, the cytoplasmic translocation of HMGB1 in hepatocytes was eliminated by MCC950 treatment. (e) Immunohistochemistry showed a high number of ASK1^+^ hepatocytes (red arrows) in old mice after chronic‐plus‐binge alcohol feeding, and this positive staining was reduced upon MCC950 treatment. (f) Assessment of plasma ALT levels. (g, h) Sirius Red staining and quantification. (i) Immunohistochemistry revealed excessive collagen I deposition in old mice after chronic‐plus‐binge alcohol feeding, and regression of liver fibrosis was observed in MCC950‐treated mice. (j) Real‐time PCR analysis for mRNA levels of pro‐fibrogenic genes, such as Col1a1 and α‐SMA. All images were acquired using 20× or 40× objectives. The data were expressed as means ± SEM, n = 6–8. *p < 0.05, **p < 0.01, or ***p < 0.001 between two groups.

Experimental and clinical ALD has been shown to be associated with high expression of NLRP3 inflammasome components (Petrasek et al., 2012). Young mice deficient in its essential components (*Casp1* ^−/−^) are significantly protected against ALD (Petrasek et al., 2012). To assess the potential role of an NLRP3 inhibitor in age‐associated liver fibrosis, we employed a recently established aging mouse model of liver fibrosis caused by chronic‐plus‐binge alcohol feeding (Ramirez et al., 2017), which is distinct from a young mouse model that exhibits chronic‐plus‐binge alcohol feeding‐induced fatty liver and liver injury without detectable fibrosis. Specifically, this aging model of liver fibrosis replicates the drinking pattern and liver fibrosis seen in older patients with ALD (Ramirez et al., 2017; Ren et al., 2022). To this end, old mice were administrated a control liquid diet, an ethanol liquid diet, or an ethanol liquid diet supplemented with a selective NLRP3 inhibitor (MCC950) (Coll et al., 2019), As illustrated in Figure 6a–c, mRNA levels of NLRP3 and IL‐1β were significantly elevated in ethanol‐fed old mice compared with control mice. An increase in the recruitment of F4/80^+^ macrophages and MPO^+^ neutrophils was also observed in ethanol‐fed old mice. These data support the concept that progressive alcoholic fibrosis during aging is associated with persistent activation of NLRP3 and recruitment of inflammatory cells, which release pro‐fibrogenic cytokines. In contrast, MCC950‐treated old mice displayed a ~50% decrease in IL‐1β expression and lowered intrahepatic infiltration of F4/80^+^ and MPO^+^ cells. In the pathophysiology of ALD, hepatic insult has been shown to trigger stressed hepatocytes to release danger‐associated molecular patterns (DAMPs), such as high‐mobility group box 1 (HMGB1) (Ge et al., 2018). The release of HMGB1 from damaged cells triggers a cascade of inflammatory cytokine/chemokine events, which further aggravates organ damage and accelerates liver fibrosis (Ge et al., 2018). The activity of HMGB1 requires translocation of HMGB1 from the nucleus to the cytoplasm and releases it into the extracellular space. Next, we asked whether NLRP3 inhibition may affect sensitivity of hepatocytes to alcohol‐triggered stress/danger signaling. As shown in Figure 6d–f, chronic‐plus‐binge ethanol feeding led to the cytoplasmic translocation of HMGB1 in hepatocytes, demonstrating increased danger signaling in ethanol‐fed old mice. Interestingly, increased ASK1^+^ hepatocytes were coupled with the cytoplasmic translocation of HMGB1, implying that ASK1 signaling from apoptotic/necrotic cells possibly promotes the passive release of HMGB1. Notably, in the absence of ALD, positive staining for HMGB1 appeared in the nucleus of hepatocytes, and ASK1^+^ cells were rarely noted. Strikingly, histological analysis showed that treatment with MCC950 reduced extranuclear translocation of HMGB1 and the number of ASK1^+^ hepatocytes and alleviated steatosis in ethanol‐fed old mice. Plasma ALT levels were increased by ~fourfold in ethanol‐fed old mice but lowered by ~60% in MCC950‐treated old mice. These results uncover that NLRP3 is required for age‐ and alcohol‐induced inflammation and liver injury.

### 4.12 Inhibition of NLRP3 by MCC950 mitigates age‐ and alcohol‐associated liver fibrosis

To determine the functional consequence of NLRP3 inhibition on liver fibrosis, Sirius Red Staining and immunohistochemical staining for collagen 1 showed perisinusoidal fibrosis in control old mice; a “chicken wire‐like” pattern of fibrosis was detected in old mice after chronic‐plus‐binge ethanol feeding (Figure 6g–i). Expression of Col1a1 and α‐SMA was significantly increased by ~threefold and ~twofold, respectively (Figure 6j), suggesting that hyperactivation of NLRP3 may promote alcoholic liver fibrosis in old mice, in agreement with the fibrotic phenotype in NLRP3 knock‐in mice (Wree et al., 2014). In contrast, MMC950 treatment downregulated fibrogenic markers and effectively attenuated age‐ and alcohol‐induced liver fibrosis (Figure 6g–j), concordant with its ability to ameliorate hepatic inflammation. These results support the feasibility that targeting NLRP3 by MCC950 modulates inflammation and reduces stressed hepatocytes, thereby reversing age‐related liver fibrosis.

## 5 DISCUSSION

This current study, along with recent studies (Ramirez et al., 2017; Ren et al., 2022), establishes an in vivo murine system of age‐ and alcohol‐induced liver injury, which mimics liver fibrosis in older patients with ALD (Ramirez et al., 2017). This preclinical mouse model enables us to investigate the mechanistic basis and potential therapeutic applications to reverse age‐associated liver fibrosis. We demonstrate, for the first time to our knowledge, that age‐dependent loss of SIRT1 is linked to activation of the NLRP3 inflammasome and perpetuates the progression of liver fibrosis. Importantly, this regulatory mechanism, which can be targeted by NLRP3 inhibition, modulates the progression of alcoholic liver fibrosis in old mice. The present study provides an attractive working model, in which age‐dependent decline of SIRT1 initiates NLRP3 activation and drives HSC activation, leading to severe fibrosis in old mice in response to toxic or alcoholic liver injury, even after injury cessation. Unlike aged mice, restoration of SIRT1 inhibits NLRP3 signaling and enhances the capacity to resolve fibrosis in young mice after injury recovery. Inhibition of NLRP3 by MCC950 ameliorates age‐ and alcohol‐induced liver fibrosis by decreasing inflammatory cell infiltration and hepatocyte‐derived stress signaling—ASK1 and HMGB1. Thus, concurrent elimination of SIRT1 and activation of NLRP3 may be considered a pathological feature of age‐associated fibrosis and a potential therapeutic target.

### 5.1 Defective SIRT1 in hepatocytes may represent a previously unrecognized mechanism for age‐related liver fibrosis

One of our most important findings is that age‐dependent susceptibility to liver fibrosis is likely attributed to impaired SIRT1 signaling in hepatocytes. Despite the identification of numerous drug candidates and extensive pre‐clinical studies, none have translated into effective treatments for patients with liver cirrhosis (Koyama & Brenner, 2017). One potential reason for the lack of clinical translation is the failure to consider the emerging concept of liver cirrhosis as an age‐related disease. Pre‐clinical animal models of liver fibrosis are largely employed in young mice that have a predominantly self‐limited fibrotic response (Jun & Lau, 2018). Therapeutic interventions are largely preventative with drug candidates before or at the time of injury, rather than curative with delayed drug administration, typically after fibrosis has been established. Our findings raise the possibility that the lack of clinical translation may not be due to the injury model per se or species differences, but rather to the context of age. An age‐related model of persistent fibrosis described in this study can be considered for evaluating drug candidates with clinical translation potential. Specifically, restoration of SIRT1 enables the liver to resolve fibrosis in young mice after cessation of liver injury. In contrast, prolonged inhibition of SIRT1 acquires a sustained inflammatory phenotype and leads to abnormal ECM remodeling that impairs the resolution of established fibrosis in old mice even after cessation of injury. We have demonstrated that genetic deletion of SIRT1 in hepatocytes exacerbates alcohol‐induced mTORC1 and fatty liver in young mice (Chen et al., 2018). Intriguingly, the role of age‐related decline in SIRT1 in promoting fibrosis is further emphasized by the fact that mice lacking SIRT1 in hepatocytes show HSC activation and develop severe fibrosis in the hepatotoxin model. SIRT1 LKO mice may be considered as a mouse model for assessing age‐related changes in liver pathologies. The present study suggests that hepatocyte‐specific loss of SIRT1 is one of the mechanisms that contribute to persistent fibrosis during aging, in addition to other cell types. Consistent with these findings, HSC‐specific deletion of SIRT1 aggravates bile duct ligation‐induced liver fibrosis in young mice (Li et al., 2018). Importantly, chronic alcohol users show a reduction in neutrophilic SIRT1 in middle‐aged patients (Ren et al., 2022). In agreement with our findings, deletion of SIRT1 in myeloid cells, including neutrophils, promotes alcohol‐induced liver injury and inflammation in mice (Ren et al., 2022). Loss‐of‐function of SIRT1 in HSCs also contributes to alcoholic liver fibrosis in vivo; overexpression of SIRT1 inhibits activation of primary HSCs isolated from middle‐aged mice by downregulating platelet‐derived growth factor receptor‐alpha (PDGFR‐α) and c‐Myc in vitro (Ramirez et al., 2017). Our studies identify SIRT1 as an endogenous regulator of the fibroblast growth factor 21 (FGF21) hepatocyte‐derived hormone FGF21 which reduces hepatic steatosis and increases energy expenditure in young mice (Li et al., 2014). Thus, we suspect that age‐dependent defects of SIRT1 accelerate the development of liver fibrosis via a mechanism involving altered FGF21. This possibility is also supported by the fact that hepatic overexpression of FGF21 in transgenic mice markedly extends lifespan (Zhang et al., 2012). It would be of interest to further define the in vivo mechanistic role of FGF21 in liver fibrosis associated with SIRT1 loss during aging.

### 5.2 Age‐dependent decline of hepatic SIRT1 drives NLRP3‐mediated inflammation and promotes liver fibrosis even after cessation of injury

The NLRP3 inflammasome is implicated in ALD progression in young mice (Choudhury et al., 2020; Petrasek et al., 2012). In the present study, prolonged inhibition of SIRT1 results in persistent activation of NLRP3 and triggers the production of pro‐inflammatory cytokines, such as IL‐1β, in age‐associated liver fibrosis. The concept that SIRT1 serves as a negative regulator of NLRP3 in hepatocytes is strengthened by the fact that deletion of SIRT1 in hepatocytes leads to NLRP3^+^ cell elevation and severe liver fibrosis in young mice, mimicking the effect of aging on NLRP3 signaling. Interestingly, the present study shows a high number of NLRP3^+^ and ASK1^+^ in damaged hepatocytes of old mice even after the discontinued injury. We postulate that during aging, aberrant activation of ASK1 and NLRP3‐mediated inflammation can trigger a positive signaling loop that exacerbates the initial toxic insult and impairs the liver's ability to resolve established fibrosis. In addition, NLRP3 activity is tightly controlled by the multilayered regulation at the transcriptional and post‐transcriptional levels, such as phosphorylation, ubiquitination, and acetylation (He et al., 2020; Song et al., 2017). This multilayered regulation can switch on NLRP3 signaling, which is critical for a robust inflammatory response. Future study is needed to define the precise mechanism for the downregulation of the NLRP3 inflammasome complex by SIRT1.

### 5.3 Inhibition of the NLRP3 inflammasome ameliorates age‐ and alcohol‐induced liver fibrosis in mice

One of our interesting findings is that inhibition of the NLRP3 inflammasome reverses established liver fibrosis in young mice after injury recovery and protects against age‐ and alcohol‐associated liver fibrosis. ALD is considered a chronic inflammatory disease, in which the potent inflammatory cytokine IL‐1β plays a causal role in disease progression (Petrasek et al., 2012). Indeed, interventions that interfere with IL‐1β signaling or its downstream pathways improve ALD outcomes in young mice (Petrasek et al., 2012). A great interest in developing small molecules that target inflammation has been sparked with the recent success of the IL‐1 antibody canakinumab in the CANTOS clinical trial, which demonstrates a lower incidence of recurrent cardiovascular events (Ridker et al., 2017). However, patients administered canakinumab had a higher incidence of fatal infections, suggesting that despite its positive effects on chronic inflammatory diseases, long‐term inhibition of this important cytokine may compromise host immune defenses (Ridker et al., 2017). Recently, the development of specific NLRP3 inhibitors has sparked great interest in anti‐inflammatory therapeutics. MCC950 is a small‐molecule NLRP3 inhibitor that specifically binds to the ATP hydrolysis or Walker B motif of NLRP3, thereby preventing NLRP3 activity and IL‐1β maturation and production (Coll et al., 2015, 2019). At present, it is unknown what effect, if any, inhibition of the NLRP3 inflammasome might have on age‐associated liver fibrosis. In our interventional approach, MCC950 treatment alleviates alcoholic liver fibrosis in old mice by reducing inflammatory cells and hepatocellular stress/danger signaling. First, NLRP3 inhibition by MCC950 effectively suppresses IL‐1β expression, inflammatory cell infiltration, and liver fibrosis in alcohol‐fed old mice. Given that IL‐1β promotes HSC differentiation into myofibroblasts (Gieling et al., 2009), it is conceivable that inhibition of IL‐1β by MCC950 may explain the therapeutic effect of NLRP3 inhibition on age‐related liver fibrosis. Second, NLRP3 inhibition may alleviate persistent fibrosis associated with aging by inhibiting hepatocyte‐derived ASK1 and HMGB1 signaling and ameliorating liver injury. MCC950‐mediated inhibition of NLRP3 protects against liver fibrosis during aging, thereby offering a therapeutic avenue with fewer side effects in the elderly.

In conclusion, the present study defines the mechanism by which age‐related SIRT1 loss induces the NLRP3 inflammatory pathway and promotes severe and persistent liver fibrosis during aging. In this era of transformative and targeted anti‐inflammatory therapies, small‐molecule NLRP3 inhibitors, such as MCC950, may offer major advances in addressing the unmet need of alcoholic liver fibrosis associated with aging.

## AUTHOR CONTRIBUTIONS

JA, QS, and SBS conducted experiments and analyzed the data; GLS, ZL, HC, MW, and QP conducted some experiments; KDA and GLS edited the manuscript; JH, MM, AK, and NM helped with data analysis and edited the manuscript; MZ contributed to conceptualization, design, and financial support of the study, supervised the whole project, and wrote the paper.

## FUNDING INFORMATION

This work was supported in part by the National Institutes of Health Grants RO1 DK100603, RO1 DK121527, and R21 AA026922 (to M.Z.). M.Z. is also supported in part by the Distinguished Chair Endowment Fund in Aging Research from the Ewing Halsell Foundation at the University of Texas Health Science Center at San Antonio.

## CONFLICT OF INTEREST STATEMENT

None declared.

## Supporting information


Appendix S1

## Data Availability

All data generated and/or analyzed during this study are included in this article, and the data that support the results of this study are available from the corresponding author upon reasonable request.

## References

[acel13811-bib-0001] Asrani, S. K. , Devarbhavi, H. , Eaton, J. , & Kamath, P. S. (2019). Burden of liver diseases in the world. Journal of Hepatology, 70(1), 151–171. 10.1016/j.jhep.2018.09.014 30266282

[acel13811-bib-0002] Chen, H. , Shen, F. , Sherban, A. , Nocon, A. , Li, Y. , Wang, H. , Xu, M. J. , Rui, X. , Han, J. , Jiang, B. , Lee, D. , Li, N. , Keyhani‐Nejad, F. , Fan, J. G. , Liu, F. , Kamat, A. , Musi, N. , Guarente, L. , Pacher, P. , … Zang, M. (2018). DEP domain‐containing mTOR‐interacting protein suppresses lipogenesis and ameliorates hepatic steatosis and acute‐on‐chronic liver injury in alcoholic liver disease. Hepatology, 68(2), 496–514. 10.1002/hep.29849 29457836 PMC6097912

[acel13811-bib-0003] Choudhury, A. , Bullock, D. , Lim, A. , Argemi, J. , Orning, P. , Lien, E. , Bataller, R. , & Mandrekar, P. (2020). Inhibition of HSP90 and activation of HSF1 diminish macrophage NLRP3 inflammasome activity in alcohol‐associated liver injury. Alcoholism, Clinical and Experimental Research, 44(6), 1300–1311. 10.1111/acer.14338 32282939 PMC7328660

[acel13811-bib-0004] Coll, R. C. , Hill, J. R. , Day, C. J. , Zamoshnikova, A. , Boucher, D. , Massey, N. L. , Chitty, J. L. , Fraser, J. A. , Jennings, M. P. , Robertson, A. A. B. , & Schroder, K. (2019). MCC950 directly targets the NLRP3 ATP‐hydrolysis motif for inflammasome inhibition. Nature Chemical Biology, 15(6), 556–559. 10.1038/s41589-019-0277-7 31086327

[acel13811-bib-0005] Coll, R. C. , Robertson, A. A. B. , Chae, J. J. , Higgins, S. C. , Muñoz‐Planillo, R. , Inserra, M. C. , Vetter, I. , Dungan, L. S. , Monks, B. G. , Stutz, A. , Croker, D. E. , Butler, M. S. , Haneklaus, M. , Sutton, C. E. , Núñez, G. , Latz, E. , Kastner, D. L. , Mills, K. H. , Masters, S. L. , … O'Neill, L. A. J. (2015). A small‐molecule inhibitor of the NLRP3 inflammasome for the treatment of inflammatory diseases. Nature Medicine, 21(3), 248–255. 10.1038/nm.3806 PMC4392179 25686105

[acel13811-bib-0006] Cox, T. R. , Bird, D. , Baker, A. M. , Barker, H. E. , Ho, M. W. , Lang, G. , & Erler, J. T. (2013). LOX‐mediated collagen crosslinking is responsible for fibrosis‐enhanced metastasis. Cancer Research, 73(6), 1721–1732. 10.1158/0008-5472.can-12-2233 23345161 PMC3672851

[acel13811-bib-0007] Crabb, D. W. , Im, G. Y. , Szabo, G. , Mellinger, J. L. , & Lucey, M. R. (2020). Diagnosis and treatment of alcohol‐associated liver diseases: 2019 practice guidance from the American Association for the Study of Liver Diseases. Hepatology, 71(1), 306–333. 10.1002/hep.30866 31314133

[acel13811-bib-0008] Ge, X. , Arriazu, E. , Magdaleno, F. , Antoine, D. J. , Dela Cruz, R. , Theise, N. , & Nieto, N. (2018). High mobility group box‐1 drives fibrosis progression signaling via the receptor for advanced glycation end products in mice. Hepatology, 68(6), 2380–2404. 10.1002/hep.30093 29774570 PMC6240507

[acel13811-bib-0009] Gieling, R. G. , Wallace, K. , & Han, Y. P. (2009). Interleukin‐1 participates in the progression from liver injury to fibrosis. American Journal of Physiology. Gastrointestinal and Liver Physiology, 296(6), G1324–G1331. 10.1152/ajpgi.90564.2008 19342509 PMC2697947

[acel13811-bib-0010] Gong, T. , Liu, L. , Jiang, W. , & Zhou, R. (2020). DAMP‐sensing receptors in sterile inflammation and inflammatory diseases. Nature Reviews Immunology, 20(2), 95–112. 10.1038/s41577-019-0215-7 31558839

[acel13811-bib-0011] Guo, H. , Callaway, J. B. , & Ting, J. P. (2015). Inflammasomes: Mechanism of action, role in disease, and therapeutics. Nature Medicine, 21(7), 677–687. 10.1038/nm.3893 PMC4519035 26121197

[acel13811-bib-0012] He, M. , Chiang, H. H. , Luo, H. , Zheng, Z. , Qiao, Q. , Wang, L. , Tan, M. , Ohkubo, R. , Mu, W. C. , Zhao, S. , Wu, H. , & Chen, D. (2020). An acetylation switch of the NLRP3 inflammasome regulates aging‐associated chronic inflammation and insulin resistance. Cell Metabolism, 31(3), 580–591.e5. 10.1016/j.cmet.2020.01.009 32032542 PMC7104778

[acel13811-bib-0013] Hou, X. , Xu, S. , Maitland‐Toolan, K. A. , Sato, K. , Jiang, B. , Ido, Y. , Lan, F. , Walsh, K. , Wierzbicki, M. , Verbeuren, T. J. , Cohen, R. A. , & Zang, M. (2008). SIRT1 regulates hepatocyte lipid metabolism through activating AMP‐activated protein kinase. The Journal of Biological Chemistry, 283(29), 20015–20026. 10.1074/jbc.M802187200 18482975 PMC2459285

[acel13811-bib-0014] Hunt, N. J. , Kang, S. W. S. , Lockwood, G. P. , Le Couteur, D. G. , & Cogger, V. C. (2019). Hallmarks of aging in the liver. Computational and Structural Biotechnology Journal, 17, 1151–1161. 10.1016/j.csbj.2019.07.021 31462971 PMC6709368

[acel13811-bib-0015] Imai, S. , & Guarente, L. (2014). NAD+ and sirtuins in aging and disease. Trends in Cell Biology, 24(8), 464–471. 10.1016/j.tcb.2014.04.002 24786309 PMC4112140

[acel13811-bib-0016] Jun, J. I. , & Lau, L. F. (2018). Resolution of organ fibrosis. The Journal of Clinical Investigation, 128(1), 97–107. 10.1172/jci93563 29293097 PMC5749507

[acel13811-bib-0017] Koyama, Y. , & Brenner, D. A. (2017). Liver inflammation and fibrosis. The Journal of Clinical Investigation, 127(1), 55–64. 10.1172/jci88881 28045404 PMC5199698

[acel13811-bib-0018] Lackner, C. , & Tiniakos, D. (2019). Fibrosis and alcohol‐related liver disease. Journal of Hepatology, 70(2), 294–304. 10.1016/j.jhep.2018.12.003 30658730

[acel13811-bib-0019] Li, M. , Hong, W. , Hao, C. , Li, L. , Wu, D. , Shen, A. , Lu, J. , Zheng, Y. , Li, P. , & Xu, Y. (2018). SIRT1 antagonizes liver fibrosis by blocking hepatic stellate cell activation in mice. The FASEB Journal, 32(1), 500–511. 10.1096/fj.201700612R 28970250

[acel13811-bib-0020] Li, Y. , Wong, K. , Giles, A. , Jiang, J. , Lee, J. W. , Adams, A. C. , Kharitonenkov, A. , Yang, Q. , Gao, B. , Guarente, L. , & Zang, M. (2014). Hepatic SIRT1 attenuates hepatic steatosis and controls energy balance in mice by inducing fibroblast growth factor 21. Gastroenterology, 146(2), 539–549.e7. 10.1053/j.gastro.2013.10.059 24184811 PMC4228483

[acel13811-bib-0021] Li, Y. , Xu, S. , Giles, A. , Nakamura, K. , Lee, J. W. , Hou, X. , Donmez, G. , Li, J. , Luo, Z. , Walsh, K. , Guarente, L. , & Zang, M. (2011). Hepatic overexpression of SIRT1 in mice attenuates endoplasmic reticulum stress and insulin resistance in the liver. The FASEB Journal, 25(5), 1664–1679. 10.1096/fj.10-173492 21321189 PMC3079300

[acel13811-bib-0022] Pawelec, G. , Goldeck, D. , & Derhovanessian, E. (2014). Inflammation, ageing and chronic disease. Current Opinion in Immunology, 29, 23–28. 10.1016/j.coi.2014.03.007 24762450

[acel13811-bib-0023] Petrasek, J. , Bala, S. , Csak, T. , Lippai, D. , Kodys, K. , Menashy, V. , Barrieau, M. , Min, S. Y. , Kurt‐Jones, E. A. , & Szabo, G. (2012). IL‐1 receptor antagonist ameliorates inflammasome‐dependent alcoholic steatohepatitis in mice. The Journal of Clinical Investigation, 122(10), 3476–3489. 10.1172/jci60777 22945633 PMC3461900

[acel13811-bib-0024] Place, D. E. , Samir, P. , Karki, R. , Briard, B. , Vogel, P. , & Kanneganti, T. D. (2018). ASK family kinases are required for optimal NLRP3 inflammasome priming. The American Journal of Pathology, 188(4), 1021–1030. 10.1016/j.ajpath.2017.12.006 29353059 PMC6436110

[acel13811-bib-0025] Ramirez, T. , Li, Y. M. , Yin, S. , Xu, M. J. , Feng, D. , Zhou, Z. , Zang, M. , Mukhopadhyay, P. , Varga, Z. V. , Pacher, P. , Gao, B. , & Wang, H. (2017). Aging aggravates alcoholic liver injury and fibrosis in mice by downregulating sirtuin 1 expression. Journal of Hepatology, 66(3), 601–609. 10.1016/j.jhep.2016.11.004 27871879 PMC5316497

[acel13811-bib-0026] Ren, R. , He, Y. , Ding, D. , Cui, A. , Bao, H. , Ma, J. , Hou, X. , Li, Y. , Feng, D. , Li, X. , Liangpunsakul, S. , Gao, B. , & Wang, H. (2022). Aging exaggerates acute‐on‐chronic alcohol‐induced liver injury in mice and humans by inhibiting neutrophilic sirtuin 1‐C/EBPα‐miRNA‐223 axis. Hepatology, 75(3), 646–660. 10.1002/hep.32152 34510484 PMC8844214

[acel13811-bib-0027] Ridker, P. M. , Everett, B. M. , Thuren, T. , MacFadyen, J. G. , Chang, W. H. , Ballantyne, C. , Fonseca, F. , Nicolau, J. , Koenig, W. , Anker, S. D. , Kastelein, J. J. P. , Cornel, J. H. , Pais, P. , Pella, D. , Genest, J. , Cifkova, R. , Lorenzatti, A. , Forster, T. , Kobalava, Z. , … Glynn, R. J. (2017). Antiinflammatory therapy with canakinumab for atherosclerotic disease. The New England Journal of Medicine, 377(12), 1119–1131. 10.1056/NEJMoa1707914 28845751

[acel13811-bib-0028] Song, N. , Liu, Z. S. , Xue, W. , Bai, Z. F. , Wang, Q. Y. , Dai, J. , Liu, X. , Huang, Y. J. , Cai, H. , Zhan, X. Y. , Han, Q. Y. , Wang, H. , Chen, Y. , Li, H. Y. , Li, A. L. , Zhang, X. M. , Zhou, T. , & Li, T. (2017). NLRP3 phosphorylation is an essential priming event for inflammasome activation. Molecular Cell, 68(1), 185–197.e6. 10.1016/j.molcel.2017.08.017 28943315

[acel13811-bib-0029] Weinlich, R. , Oberst, A. , Beere, H. M. , & Green, D. R. (2017). Necroptosis in development, inflammation and disease. Nature Reviews. Molecular Cell Biology, 18(2), 127–136. 10.1038/nrm.2016.149 27999438

[acel13811-bib-0030] Wree, A. , Eguchi, A. , McGeough, M. D. , Pena, C. A. , Johnson, C. D. , Canbay, A. , Hoffman, H. M. , & Feldstein, A. E. (2014). NLRP3 inflammasome activation results in hepatocyte pyroptosis, liver inflammation, and fibrosis in mice. Hepatology, 59(3), 898–910. 10.1002/hep.26592 23813842 PMC4008151

[acel13811-bib-0031] Yang, L. , Inokuchi, S. , Roh, Y. S. , Song, J. , Loomba, R. , Park, E. J. , & Seki, E. (2013). Transforming growth factor‐β signaling in hepatocytes promotes hepatic fibrosis and carcinogenesis in mice with hepatocyte‐specific deletion of TAK1. Gastroenterology, 144(5), 1042–1054.e4. 10.1053/j.gastro.2013.01.056 23391818 PMC3752402

[acel13811-bib-0032] Zhang, Y. , Xie, Y. , Berglund, E. D. , Coate, K. C. , He, T. T. , Katafuchi, T. , Xiao, G. , Potthoff, M. J. , Wei, W. , Wan, Y. , Yu, R. T. , Evans, R. M. , Kliewer, S. A. , & Mangelsdorf, D. J. (2012). The starvation hormone, fibroblast growth factor‐21, extends lifespan in mice. eLife, 1, e00065. 10.7554/eLife.00065 23066506 PMC3466591

