# Supplementary material for: Age‐dependent loss of hepatic SIRT1 enhances NLRP3 inflammasome signaling and impairs capacity for liver fibrosis resolution
Source: Aging Cell. 2023 Mar 31;22(5):e13811. doi: 10.1111/acel.13811 (PMC10186605; doi:10.1111/acel.13811)
Supplement: Supplementary file 1 — Appendix S1 [file ACEL-22-e13811-s001.pdf]

Age-Dependent Loss of Hepatic SIRT1 Enhances NLRP3 Inflammasome Signaling and Impairs Capacity for Liver Fibrosis Resolution

Short Title—Interplay of SIRT1 and NLRP3 in Aging Liver

Jennifer Adjei-Mosi[1,2], Qing Sun[1,2], Steven Blake Smithson[1,2], Gavyn Lee Shealy[1,2], Krupa Dhruvitha Amerineni[1,2], Zerong Liang[1,2], Hanqing Chen[1,2], Mei Wang[1,2], Qinggong Ping[1,2], Jingyan Han[3], Masahiro Morita[1,2], Amrita Kamat[4], Nicolas Musi[2,4], Mengwei Zang[1,2,4]*

Animal Care

Mice were housed in a specific-pathogen-free facility with sterile caging on ventilated cage racks that deliver HEPA-filtered air to each cage with free access to acidified RO water bottles. The housing for the room described was equipped with sterilized equipment. Mice were housed in a temperature-controlled environment with 12:12 light: dark cycle and fed the Envigo 7912 Traditional Rodent Diet (Envigo, Greenfield, IN). There was a maximum of four (male) and five (female) mice per cage. The housing was in a non-transient room where only authorized personnel who had not been in other transient rooms were allowed to enter. Husbandry and animal care were provided by experienced animal care staff available 24/7 and veterinarians weekly. The veterinarian or animal care staff recommended euthanasia if mice had any health issues, such as wounds from fighting, severe dermatitis, tumors, or other signs of morbidity. These unhealthy mice were excluded from the study. Hepatocyte-specific SIRT1 knockout (SIRT1 LKO) mice on C57BL/6 background as well as their wild-type control mice, which were described previously (Chen et al., 2018; Li et al., 2014), were both bred in our facility. C57BL/6 mice were originally obtained from the Jackson Laboratory, then bred in our facility, and given the Envigo 7904 Breeding Traditional Rodent Diet (Envigo, Greenfield, IN). All animal experiments were approved by the Institutional Animal Care and Use Committee (IACUC) at the University of Texas Health San Antonio (UTHSA).

Young and old mouse models of CCl_4-induced liver fibrosis

To evaluate the reparative response to liver injury in young and old mice, a mouse model of CCl_4-induced liver fibrosis was used as described previously (Ramirez et al., 2017; Ren et al., 2022; L. Yang et al., 2013; Y. M. Yang et al., 2019). Mice at 4 to 6 months of age (young) and mice at 16 to 20 months of age (old) were intraperitoneally injected with CCl_4 (Cat#319961, Sigma-Aldrich) diluted 1:4 in olive oil at a dose of 0.5 µL/g body weight twice weekly for 4 weeks with a total of 8 injections. The two age-matched control groups were conducted with repeated olive oil injections. To evaluate dynamic changes in liver fibrosis during the two distinct phases of the CCl_4 model, the initial liver injury phase and subsequent recovery phase, mice were sacrificed at different time points after the last dose of CCl_4. For the fibrosis phase, the mice were sacrificed 24 hours after the final injection. For the recovery phase, the mice were sacrificed 5 days after the final injection, giving time for the mice to recover from CCl_4-induced liver injury. The body weight of the mice will be measured. The percentage change in body weight was expressed as a percentage change in body weight measurements of the mice between the initial and the final day of the experiment. All mice were housed in a temperature-controlled environment with a 12-hour dark/12-hour light cycle. All animal experiments were approved by the Institutional Animal Care and Use Committee (IACUC) at the University of Texas Health San Antonio.

Hepatocyte-specific SIRT1 knockout (SIRT1 LKO) mice with chronic liver injury

SIRT1 floxed mice that contain the deletion of SIRT1 exon 4, which encodes 51 amino acids of the conserved catalytic domain of SIRT1, were crossed with Albumin (Alb)-Cre transgenic mice to generate hepatocyte-specific SIRT1 knockout (SIRT1 LKO) mice as described previously (Chen et al., 2018; Li et al., 2014). We used Alb-Cre negative *SIRT1* floxed mice as age-matched Wild-type (WT) littermate controls. SIRT1 LKO mice and their WT littermates at 3- to 5- months of age were intraperitoneally injected with either olive oil or CCl_4 at the dose of 0.5 μL/g body weight twice weekly for 6 weeks with a total of 12 injections. The mice were sacrificed 24 hours after their final injection.

An aging mouse model of alcohol-induced liver injury

The experimental old mouse model of chronic-plus-binge ethanol feeding-induced liver injury and fibrosis was recently developed by Bin Gao's group at NIH/NIAAA (Adeline Bertola, Mathews, Ki, Wang, & Gao, 2013; A. Bertola, Park, & Gao, 2013). Old mice (15-18 months) were initially fed the Lieber-DeCarli control liquid diet (Cat#F1259SP, Bio-Serv, Frenchtown, NJ) *ad libitum* for 5 days during acclimatization. The ethanol-fed group was subsequently allowed free access to a Lieber-DeCarli alcohol liquid diet (Cat# F1258SP, Bio-Serv, Frenchtown, NJ) containing 5% (v/v) ethanol for 10 days. On day 11, the mice received a single dose of ethanol (5 g/kg body weight) via oral gavage in the early morning and were sacrificed 9 hours later. Pair-fed mice were placed with an isocaloric control diet for 10 days and were given an oral gavage with isocaloric dextrin-maltose in the early morning of day 11. They were sacrificed 9 hours later.

MCC950 treatment in an aging mouse model of alcohol-induced liver fibrosis

To determine the effect of MCC950, a selective NLRP3 inhibitor, on the development of alcoholic fibrosis, old mice (15-18 months) were treated with MCC950 (Cat#HY-12815, MedChemExpress, Monmouth Junction, NJ) in the Lieber-DeCarli alcohol liquid diet containing MCC950 at a dose of 20 mg/kg/day (Coll et al., 2015). After 5 days of acclimatization, administration of MCC950 started on the first day of ethanol feeding and continued for 10 days until the end of the experiment. These MCC950-treated mice were sacrificed 9 hours on day 11 following a single binge of ethanol (5 g/kg) via oral gavage. All mice were weighed daily from the beginning of the feeding period until the sacrifice date. When mice were euthanized, one portion of liver tissue was taken rapidly, freshly frozen in liquid nitrogen, and stored at -80°C until needed for real-time PCR analysis. The remaining liver tissue was fixed for histological and immunohistochemistry analyses.

Biochemical assays

Plasma alanine aminotransferase (ALT) levels in mice were determined using ALT (SGPT)- IFCC liquid reagent set (Cat# A524-150, Teco Diagnostics, Anaheim, CA) according to the manufacturer's instruction.

Liver histology

Liver tissues were rapidly fixed in phosphate-buffered 10% formalin (Cat#SF100-20, Fisher Chemical, Fair Lawn, NJ) at room temperature and embedded in paraffin while animals were sacrificed. Paraffin sections (4 μm) were cut and mounted on glass slides. After dehydration, the sections were stained with hematoxylin and eosin as described previously (Li, Xu, Giles, et al., 2011; Li, Xu, Mihaylova, et al., 2011; Luo et al., 2016).

Picrosirius red staining

To assess morphological and fibrotic changes, Picrosirius Red staining was performed in 4-μm thick cross-sections of liver tissues. Liver specimens were fixed in 10% buffered formalin and embedded in paraffin. Following deparaffinization and rehydration, liver sections were stained by Weight's Hematoxylin (Cat# S216b, Poly Scientific, Bay Shore, NY) for 10 min at room temperature and rinsed three times with distilled water. The sections were quickly dipped in 1% hydrochloric acid containing 70 % ethanol. The sections were then stained with 0.01 % (w/v) Fast Green FCF (Cat# F99-10, Fisher Scientific, Pittsburgh, PA) in 1.0 % Acetic Acid solution (Cat# 26367-07, Electron Microscopy Sciences) for 10 min at room temperature and any unbound dye was removed. After that, the sections were stained with 0.1 % (w/v) Picrosirius Red in a saturated aqueous solution of 1.0 % Picric Acid (Cat# S2365, Poly Scientific, Bay Shore, NY) for 60 min at room temperature. Sections were quickly differentiated in 1 % acetic acid solution to remove any unbound dye. After the slides were dehydrated and cleared in xylene, the sections were mounted. The Picrosirius Red stains were visualized under a Nikon Eclipse 80i microscope with 10X and 20X objectives and stained images were captured and digitalized using a Nikon DS-Fi1 digital camera attached to the Nikon Eclipse 80i microscope. To quantify Sirius Red staining, images of five or six random fields of liver sections from each mouse were calculated with NIH ImageJ software. Collagen values were expressed as the percentage of positively stained areas relative to the total areas of liver sections.

Immunohistochemistry

Fixing of liver tissue sections was performed as described previously (Li, Xu, Giles, et al., 2011; Li, Xu, Jiang, Cohen, & Zang, 2013; Luo et al., 2016). After deparaffinization and rehydration, 4-μm thick liver sections were incubated with 10 mmol/L citric acid (pH 6.0) containing 0.1% Tween and heated in a microwave (Model #MW8775W, Emerson Household Microwave Oven, Parsippany, NJ) three times to unmask the antigenicity. Sections were heated at power level 10 for 5 min, power level 6 for 2 min, and power level 4 for 2 min. After that, sections were cooled at room temperature for at least 30 min. Liver sections were incubated in 3% H_2O_2 containing 10% methanol to quench peroxidase activity for 30 min at 37°C, rinsed once with distilled water, and washed with phosphate-buffered saline (PBS, pH 7.4) containing 0.1% Tween-20 (PBST) three times for 5 min each. Nonspecific binding was blocked with 2.5 % normal horse serum (Cat# MP-7401, Vector Laboratories, Burlingame, CA) for 1 h at room temperature. For immunohistochemistry staining, the liver sections were placed in a wet chamber and incubated with the primary antibody diluted in PBST containing 1% BSA at 4°C overnight. The following primary antibodies were used: Collagen I (Cat# A16891), a-SMA (Cat# A7248), and HMGB1 (Cat# A2553) (Abclonal Technology, Woburn, MA); TGF-β1 (Cat# 3711S), NLRP3 (Cat#15101S), ASK1 (Cat#3762) (Cell Signaling Technology, Danvers, MA); F4/80 (Cat# sc-25830, Santa Cruz Biotechnology, Dallas, TX); and MPO (Cat# PP 023 AA) (Biocare Medical, Concord, CA). After the liver sections were rinsed with PBST three times for 5 min each, sections were incubated for 1 h at room temperature with the HRP-conjugated secondary antibody from the ImmPRESS® HRP Horse Anti-Rabbit IgG Polymer Detection Kit (Cat# MP-7401, Vector Laboratories, Burlingame, CA). For color development, sections were incubated with a DAB reaction product from the SignalStain® DAB Substrate Kit (Cat#8059, Cell Signaling Technology, Danvers, MA) according to the manufacturer's instructions. All positive staining was visualized by comparing it with negative controls. Finally, the liver sections were counterstained with hematoxylin, differentiated with 0.5% hydrochloric acid in 70% ethanol, cleared with xylene, and mounted. After mounting, positive staining was observed under a Nikon Eclipse 80i microscope with 20X and 40X objectives. Stained images were captured and digitalized using a Nikon DS-Fi1 digital camera attached to the Nikon Eclipse 80i microscope.

Total RNA isolation and reverse transcription-quantitative PCR (RT-qPCR)

Total RNA from liver tissues was extracted using TRIzol Reagent (Cat#15-596-026, Thermo Fisher, Waltham, MA) according to the manufacturer's instructions. Quantification of total RNA was performed in a Spectramax M5 Microplate Reader (Molecular Devices, Sunnyvale, CA, USA) following the manufacturer's instruction. One microgram of total RNA was reverse-transcribed into complementary DNA (cDNA) using a High Capacity cDNA Reverse Transcription kit (Cat# 43-688-14, Applied Biosystems, Thermo Fisher, Waltham, MA) according to the manufacturer's instructions. The cDNA synthesis was carried out in a Bio-Rad T100 Thermal Cycler (Bio-Rad, Hercules, CA). The mRNA levels were quantified by real-time PCR analysis with SYBR Green PCR master mix (Cat# A25742, Applied Biosystems, Thermo Fisher, Waltham, MA) and gene-specific primers in the Bio-Rad CFX384 Touch Real-Time PCR Detection System (Bio-Rad, Hercules, CA). The specificity of the PCR amplification was verified by melting curve analysis of the final products. The $2^{-\Delta\Delta Ct}$ method was used to calculate the relative gene expression. The relative mRNA levels of genes were normalized to those of GAPDH and expressed as relative levels to the control mice (Li et al., 2014; Li, Wong, Walsh, Gao, & Zang, 2013). The primers used for RT-qPCR are as follows:

Gene name	Forward primers	Reverse primer sequences
**SIRT1**	5'-AAACATGGCTTGAGGGTCTG-3'	5'-TCTCCTGTGGGATTCCTG AC-3'
**Col1a1**	5'-TGCTGGTCCCAAAGGTTC-3'	5'-CAGGGCGACCATCTTGAC-3'
**α-SMA**	5'-TCCCAGACATCAGGGAGTAA-3'	5'-TCGGATACTTCAGCGTCAGGA-3'
**TGF-β1**	5'-CGGGTTGTGTTGGTTGTAG-3'	5'-AAGGACCTGGGTTGGAAG-3'
**MMP-9**	5'-CCCAAAGACCTGAAAACCTC-3'	5'-ACTGCTTCTCTCCCATCATC-3'
**MMP-12**	5'-TGGTGGACTGCTAGGTTT TG-3'	5'-TCGCCTCTCTGCTGATGAC-3'
**TIMP-1**	5'-ATCTGGCATCCTCTTGTTG -3'	5'-CGCTGGTATAAGGTGGTCTC-3'
**LOX**	5'-TCCGCAAAGAGTGAAGAACC	CATCAAGCAGGTCATAGTGG
**NLRP3**	5'-AACCAATGCGAGATCCTGAC-3'	5'-GCTCTGCAACCTCCAGAAAC-3'
**TNF-α**	5'-CGTCAGCCGATTTGCTATCT-3'	5'-CGGACTCCGCAAAGTCTAAG-3'
**IL-1β**	5'-TTCGTGAATGAGCAGACAGC-3'	5'-GGTTTCTTGTGACCCTGAGC-3'
**MCP-1**	5'-CAGCCAGATGCAGTTAACGC-3'	5'-GCCTACTCATTGGGATCATCTTG-3'
**GAPDH**	5'-TGCGACTTCAACAGCAACTC-3'	5'-CTTGCTCAGTGTCCTTGCTG-3'

Statistical analysis

All statistical analyses were performed using GraphPad Prism 6.0 software (GraphPad Software, La Jolla, CA). If the datasets include two groups, a two-tailed Student's t-test was used to assess significance between the groups. If datasets contain more than two groups, one-way ANOVA followed by Tukey's post hoc test was used to assess significant differences. All data are presented as mean ± standard error of the mean (S.E.M). For consistency in comparisons, significance in all figures is denoted as follows: *$P<0.05$, **$P<0.01$, ***$P<0.001$, ****$P<0.0001$.

References

Bertola, A., Mathews, S., Ki, S. H., Wang, H., & Gao, B. (2013). Mouse model of chronic and binge ethanol feeding (the NIAAA model). *Nat. Protocols, 8*(3), 627-637.

Bertola, A., Park, O., & Gao, B. (2013). Chronic plus binge ethanol feeding synergistically induces neutrophil infiltration and liver injury in mice: a critical role for E-selectin. *Hepatology, 58*(5), 1814-1823. doi:10.1002/hep.26419

Chen, H., Shen, F., Sherban, A., Nocon, A., Li, Y., Wang, H., . . . Zang, M. (2018). DEP domain-containing mTOR-interacting protein suppresses lipogenesis and ameliorates hepatic steatosis and acute-on-chronic liver injury in alcoholic liver disease. *Hepatology, 68*(2), 496-514. doi:10.1002/hep.29849

Coll, R. C., Robertson, A. A. B., Chae, J. J., Higgins, S. C., Muñoz-Planillo, R., Inserra, M. C., . . . O'Neill, L. A. J. (2015). A small-molecule inhibitor of the NLRP3 inflammasome for the treatment of inflammatory diseases. *Nature Medicine, 21*(3), 248-255. doi:10.1038/nm.3806

Li, Y., Wong, K., Giles, A., Jiang, J., Lee, J. W., Adams, A. C., . . . Zang, M. (2014). Hepatic SIRT1 attenuates hepatic steatosis and controls energy balance in mice by inducing fibroblast growth factor 21. *Gastroenterology, 146*(2), 539-549 e537. doi:10.1053/j.gastro.2013.10.059

Li, Y., Wong, K., Walsh, K., Gao, B., & Zang, M. W. (2013). Retinoic Acid Receptor beta Stimulates Hepatic Induction of Fibroblast Growth Factor 21 to Promote Fatty Acid Oxidation and Control Whole-body Energy Homeostasis in Mice. *Journal of Biological Chemistry, 288*(15), 10490-10504.

Li, Y., Xu, S., Giles, A., Nakamura, K., Lee, J. W., Hou, X., . . . Zang, M. (2011). Hepatic overexpression of SIRT1 in mice attenuates endoplasmic reticulum stress and insulin resistance in the liver. *FASEB J, 25*(5), 1664-1679. doi:10.1096/fj.10-173492

Li, Y., Xu, S., Jiang, B., Cohen, R. A., & Zang, M. (2013). Activation of sterol regulatory element binding protein and NLRP3 inflammasome in atherosclerotic lesion development in diabetic pigs. *Plos One, 8*(6), e67532. doi:10.1371/journal.pone.0067532

Li, Y., Xu, S., Mihaylova, M. M., Zheng, B., Hou, X., Jiang, B., . . . Zang, M. (2011). AMPK phosphorylates and inhibits SREBP activity to attenuate hepatic steatosis and atherosclerosis in diet-induced insulin-resistant mice. *Cell Metab, 13*(4), 376-388. doi:10.1016/j.cmet.2011.03.009

Luo, T., Nocon, A., Fry, J., Sherban, A., Rui, X., Jiang, B., . . . Zang, M. (2016). AMPK Activation by Metformin Suppresses Abnormal Extracellular Matrix Remodeling in Adipose Tissue and Ameliorates Insulin Resistance in Obesity. *Diabetes, 65*(8), 2295-2310. doi:10.2337/db15-1122

Ramirez, T., Li, Y. M., Yin, S., Xu, M. J., Feng, D., Zhou, Z., . . . Wang, H. (2017). Aging aggravates alcoholic liver injury and fibrosis in mice by downregulating sirtuin 1 expression. *J Hepatol, 66*(3), 601-609. doi:10.1016/j.jhep.2016.11.004

Ren, R., He, Y., Ding, D., Cui, A., Bao, H., Ma, J., . . . Wang, H. (2022). Aging exaggerates acute-on-chronic alcohol-induced liver injury in mice and humans by inhibiting neutrophilic sirtuin 1-C/EBPα-miRNA-223 axis. *Hepatology, 75*(3), 646-660. doi:10.1002/hep.32152

Yang, L., Inokuchi, S., Roh, Y. S., Song, J., Loomba, R., Park, E. J., & Seki, E. (2013). Transforming growth factor-β signaling in hepatocytes promotes hepatic fibrosis and carcinogenesis in mice with hepatocyte-specific deletion of TAK1. *Gastroenterology, 144*(5), 1042-1054.e1044. doi:10.1053/j.gastro.2013.01.056

Yang, Y. M., Noureddin, M., Liu, C., Ohashi, K., Kim, S. Y., Ramnath, D., . . . Seki, E. (2019). Hyaluronan synthase 2-mediated hyaluronan production mediates Notch1 activation and liver fibrosis. *Science Translational Medicine, 11*(496), 15. doi:10.1126/scitranslmed.aat9284

Figure S1.

Adjei-Mosi J, et al.

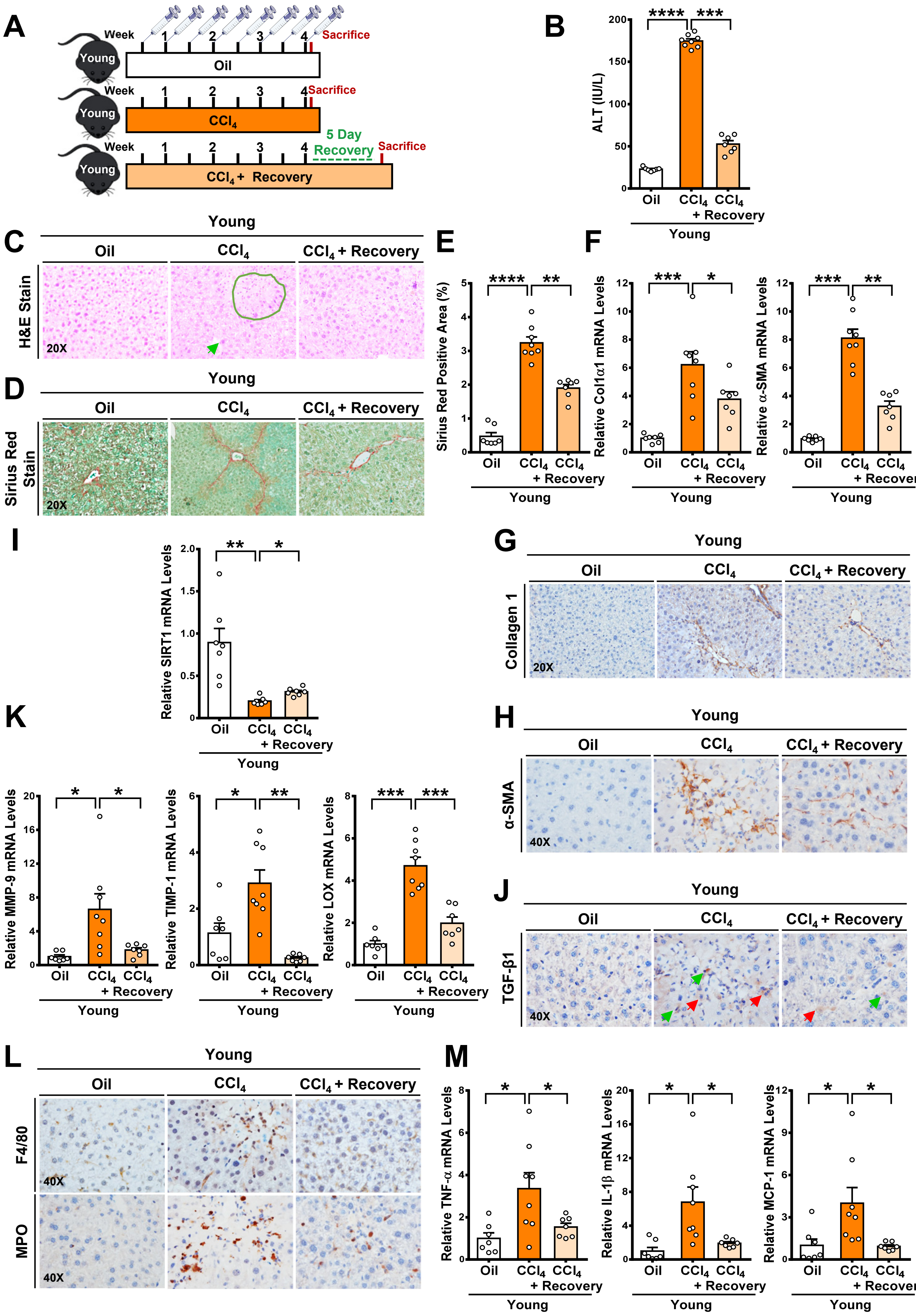

Figure S1. Fibrosis regression in young mice after cessation of liver injury is associated with SIRT1 restoration and inflammation reduction. A. Diagram illustrating the time course of both liver fibrosis and resolution of established fibrosis in young mice in the CCl_4 model. **B.** Assessment of plasma ALT levels. **C.** Representative H&E staining of liver sections in oil-injected young mice (Oil), in young mice 24 hours post-injection (CCl_4), and in young mice 5 days after the last CCl_4 injection (CCl_4 + Recovery). Hepatocyte necrosis (green outline) and inflammation (green arrows) were detected in young mice after repeated liver injury but reduced after the recovery period. **D-E.** Hepatic fibrosis was assessed by Sirius Red staining and quantified by measuring positively stained areas of Sirius Red staining. Hepatic fibrosis was increased in young mice in response to repeated liver injury and partially reduced after 5 days of recovery. **F.** Real-time PCR analysis showed that mRNA levels of the pro-fibrogenic genes, such as Col1a1 and α-SMA, were increased by CCl_4 administration and diminished after injury recovery in young mice. **G-H.** Immunohistochemistry for collagen I **(G)** and α-SMA **(H)** showed collagen deposition and accumulation of α-SMA$^+$ HSC in young mice after CCl_4 administration; clearance of activated HSCs was seen after injury recovery. **I.** Real-time PCR analysis showed that expression of SIRT1 in young mice was significantly downregulated by CCl_4 administration and partially restored after injury recovery. **J.** Immunohistochemistry showed strong staining for TGF-β1 in hepatocytes (red arrows) and other cells (green arrows) in young mice injected with CCl_4, and this elevation was eliminated upon cessation of CCl_4 injection. **K.** Real-time PCR analysis showed that expression of ECM regulators, such as MMP-9, TIMP-1, and LOX, was upregulated in young mice injected with CCl_4 but was returned to normal levels upon cessation of CCl_4 injection. **L.** Immunohistochemistry for F4/80, a macrophage marker, and MPO, a neutrophil marker. **M.** Real-time PCR analysis showed that expression of pro-*inflammatory* regulators, such as TNF-α, IL-1β, and MCP-1, was upregulated by CCl_4 injection and downregulated upon cessation of liver injury. All images were acquired using 20X or 40X objectives. The data were expressed as means ± SEM, n=6-8. *P < 0.05, **P < 0.01, *** P < 0.001, or **** P < 0.0001 between two groups.
